# Cognitive Impairment Induced by Delta9-tetrahydrocannabinol Occurs through Heteromers between Cannabinoid CB_1_ and Serotonin 5-HT_2A_ Receptors

**DOI:** 10.1371/journal.pbio.1002194

**Published:** 2015-07-09

**Authors:** Xavier Viñals, Estefanía Moreno, Laurence Lanfumey, Arnau Cordomí, Antoni Pastor, Rafael de La Torre, Paola Gasperini, Gemma Navarro, Lesley A. Howell, Leonardo Pardo, Carmen Lluís, Enric I. Canela, Peter J. McCormick, Rafael Maldonado, Patricia Robledo

**Affiliations:** 1 Neuropharmacology Laboratory, University Pompeu Fabra, Barcelona, Spain; 2 Centro de Investigación Biomédica en Red sobre Enfermedades Neurodegenerativas, Barcelona, Spain; 3 Department of Biochemistry and Molecular Biology, Faculty of Biology, University of Barcelona, Barcelona, Spain; 4 CPN, INSERM UMR S894, Université Paris Descartes, UMR S894, Paris, France; 5 Laboratori de Medicina Computacional, Unitat de Bioestadística, Facultat de Medicina, Universitat Autònoma de Barcelona, Bellaterra, Spain; 6 Integrative Pharmacology and Systems Neuroscience, IMIM-Hospital del Mar Medical Research Institute, Barcelona, Spain; 7 School of Pharmacy, University of East Anglia, Norwich Research Park, Norwich, United Kingdom; Mount Sinai School of Medicine, UNITED STATES

## Abstract

Activation of cannabinoid CB1 receptors (CB_1_R) by delta9-tetrahydrocannabinol (THC) produces a variety of negative effects with major consequences in cannabis users that constitute important drawbacks for the use of cannabinoids as therapeutic agents. For this reason, there is a tremendous medical interest in harnessing the beneficial effects of THC. Behavioral studies carried out in mice lacking 5-HT_2A_ receptors (5-HT_2A_R) revealed a remarkable 5-HT_2A_R-dependent dissociation in the beneficial antinociceptive effects of THC and its detrimental amnesic properties. We found that specific effects of THC such as memory deficits, anxiolytic-like effects, and social interaction are under the control of 5-HT_2A_R, but its acute hypolocomotor, hypothermic, anxiogenic, and antinociceptive effects are not. In biochemical studies, we show that CB_1_R and 5-HT_2A_R form heteromers that are expressed and functionally active in specific brain regions involved in memory impairment. Remarkably, our functional data shows that costimulation of both receptors by agonists reduces cell signaling, antagonist binding to one receptor blocks signaling of the interacting receptor, and heteromer formation leads to a switch in G-protein coupling for 5-HT_2A_R from Gq to Gi proteins. Synthetic peptides with the sequence of transmembrane helices 5 and 6 of CB_1_R, fused to a cell-penetrating peptide, were able to disrupt receptor heteromerization in vivo, leading to a selective abrogation of memory impairments caused by exposure to THC. These data reveal a novel molecular mechanism for the functional interaction between CB_1_R and 5-HT_2A_R mediating cognitive impairment. CB_1_R-5-HT_2A_R heteromers are thus good targets to dissociate the cognitive deficits induced by THC from its beneficial antinociceptive properties.

## Abstract 2

Specific subtypes of cannabinoid and serotonin receptors interact to form heterodimers that mediate the cognitive deficits—but not the analgesic effects—induced by tetrahydrocannabinol, the major psychoactive constituent in *Cannabis sativa*.

## Author Summary

Delta-9-tetrahydrocannabinol (THC), the main psychoactive compound of marijuana, induces numerous undesirable effects, including memory impairments, anxiety, and dependence. Conversely, THC also has potentially therapeutic effects, including analgesia, muscle relaxation, and neuroprotection. However, the mechanisms that dissociate these responses are still not known. Using mice lacking the serotonin receptor 5-HT_2A_, we revealed that the analgesic and amnesic effects of THC are independent of each other: while amnesia induced by THC disappears in the mutant mice, THC can still promote analgesia in these animals. In subsequent molecular studies, we showed that in specific brain regions involved in memory formation, the receptors for THC and the 5-HT_2A_ receptors work together by physically interacting with each other. Experimentally interfering with this interaction prevented the memory deficits induced by THC, but not its analgesic properties. Our results highlight a novel mechanism by which the beneficial analgesic properties of THC can be dissociated from its cognitive side effects.

## Introduction

The administration of delta-9-tetrahydrocannabinol (THC), the main psychoactive compound in *Cannabis sativa*, induces numerous behavioral responses related to undesirable effects, including memory impairments [1,2], anxiogenic effects [3], and dependence [4,5]. However, other effects of THC are associated with potential therapeutic applications, including analgesia [6] and anxiolytic-like and neuroprotective effects [3,5,7]. One major challenge in the field of cannabinoids is to identify new mechanisms that could be used to dissociate these responses in order to improve the benefit-risk ratio of cannabinoid agonists. Cannabinoid behavioral effects are mainly due to the activation of central CB_1_ cannabinoid receptors (CB_1_R) [5]. CB_1_R activation has been shown to modulate a wide range of neurotransmitters in the brain, including glutamate, γ-aminobutyric acid (GABA), opioids, dopamine, and serotonin, which could participate in THC pharmacological responses [4,5]. Recent evidence shows that THC and other cannabinoids modulate behaviors typically mediated by serotonin 2A receptors’ (5-HT_2A_R) activation [8–10]. In addition, mice lacking CB_1_R exhibit a dysregulation of serotonergic activity in the prefrontal cortex [11] and reduced head twitches induced by the 5-HT_2A_R agonist, (±)-1-(2,5-dimethoixy-4-odophenyl)-2-aminopropane (DOI) [12]. Reciprocally, the activation of 5-HT_2A_R expressed in cells stimulates the formation and release of the endocannabinoid, 2-arachidonoylglycerol (2-AG) [13,14]. Both 5-HT_2A_R and CB_1_R are expressed in brain structures involved in regulating emotions, learning, and memory, including the amygdala, cerebral cortex, and hippocampus [15–17]. CB_1_Rs are highly expressed presynaptically in the prefrontal cortex [18–20] and in the hippocampus [21], where moderate expression of 5-HT_2A_R has also been observed [16,22,23]. In the rat striatum, CB_1_R expression has been detected in dendrites of spiny- and aspiny-type somata [24], coinciding with the observed dendritic expression of 5-HT_2A_R in this area [25–27]. Moreover, 5-HT_2A_R is involved in different psychotic manifestations [28,29], while adolescent consumption of cannabis enhances the incidence of psychotic symptoms [30,31]. Collectively, these data suggest possible bidirectional interactions between CB_1_R- and 5-HT_2A_R-mediated pharmacological responses, although a cellular mechanism for this cross talk has yet to be discovered. Here we sought to understand at what level the interactions of these two systems occur. Using a variety of in vivo and in vitro assays, we reveal a new molecular mechanism by which the cognitive deficits of THC can be dissociated from its beneficial antinociceptive properties.

Using transgenic mice lacking 5-HT_2A_R, we found that these two receptors do indeed interact in heteromeric complexes. Importantly, these heteromers are specifically required for the amnesic, anxiolytic, and social interaction effects caused by THC, but not for other pharmacological responses, such as antinociception and hypolocomotion. Interestingly, the activation of this complex in cells expressing both receptors resulted in a reduction of intracellular signaling through adenylate cyclase, arrestin recruitment, and the extracellular signal-regulated kinase (ERK) 1/2 and protein kinase B (Akt) pathways, confirming altered downstream signaling. The formation of this receptor complex in the brain and its selective involvement in THC-induced memory and anxiety-like responses was further evidenced by the use of transmembrane helix (TM) 5 and TM 6 interference peptides in vivo. Hence, the disruption of the CB_1_R-5-HT_2A_R heteromer by intracerebroventricular (ICV) infusion of these peptides abolished the memory deficits induced by THC and its anxiolytic-like effects, but not its antinociceptive properties. Our findings reveal a new molecular mechanism by which the cognitive deficits of THC can be dissociated from its beneficial antinociceptive properties.

## Results

### 5-HT_2A_R Modulates THC’s Effects on Acute Amnesia, Anxiety, and Social Interaction and Its Effect on Dorsal Raphe Neuronal Activity

**Fig 1:**
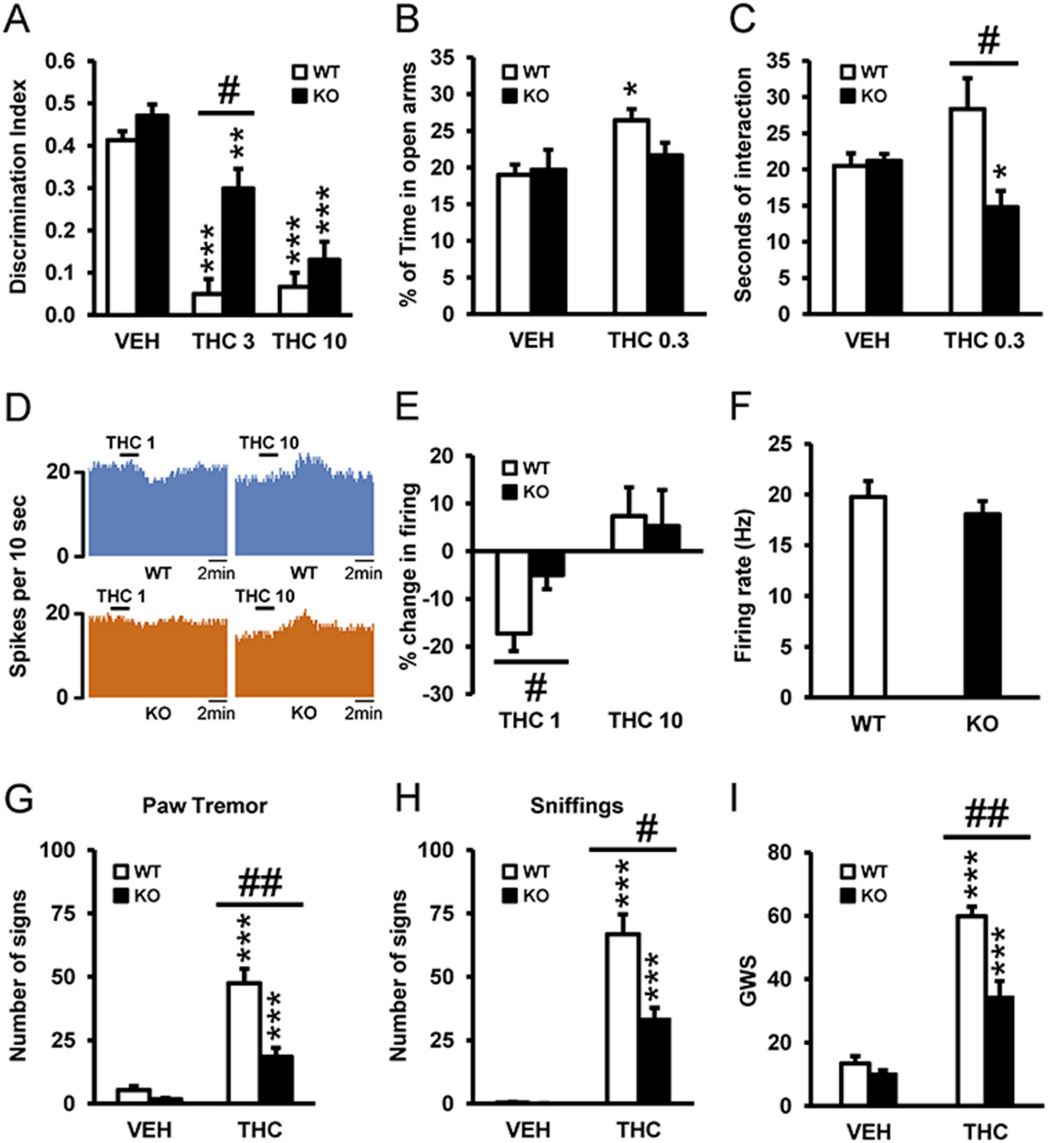
5-HT_2A_R mediates THC-induced amnesic- and anxiolytic-like effects. (A) The administration of THC (3 and 10 mg/kg) induced memory impairments in the novel object recognition test in WT mice as compared to vehicle (VEH) treatment (*n* = 8–11), and this effect was significantly abrogated in 5-HT_2A_R KO mice at the dose of 3 mg/kg, but not at the dose of 10 mg/kg. (B) The anxiolytic effects of THC (0.3 mg/kg) observed in WT mice tested in the elevated plus maze were blocked in 5-HT_2A_R KO mice (*n* = 9–12). (C) The increase in social interaction induced by THC (0.3 mg/kg) in WT mice was abolished in 5-HT_2A_R KO mice (*n* = 5–7). (D) Neuronal firing of representative dorsal raphe (DR) neurons before and after THC administration (1 and 10 nM) in WT (upper panel) and 5-HT_2A_R KO (lower panel) animals. (E) A challenge with THC (1 nM) reduced the percent change in firing rate of DR neurons from WT mice, and this effect was blunted in DR neurons from 5-HT_2A_R KO mice. No significant differences between genotypes were observed following a challenge with THC at 10 nM (*n* = 7–14). (F) The basal firing rate of DR neurons was similar in WT and 5-HT_2A_R KO animals (*n* = 20–24). (G–I) Withdrawal symptoms, including paw tremor (G), sniffing (H), and global withdrawal score (GWS) (I), were reduced in 5-HT_2A_R KO mice when compared to WT mice. Data are mean + standard error of the mean (SEM). * *p* < 0.05, ** *p* < 0.01, and *** *p* < 0.001 versus VEH; # *p* < 0.05 versus WT mice. The statistical analyses used and their corresponding F and *p*-values are shown in S1 Table.

Due to the compelling data that 5-HT_2A_R may interact with CB_1_R, we sought to broadly identify which behavioral effects of THC might be dependent on 5-HT_2A_R. Using wild-type (WT) and 5-HT_2A_R knockout (KO) animals, we evaluated THC’s amnesic-like effects at two doses (3 and 10 mg/kg) known to induce memory deficits in the novel object recognition paradigm [1]. Acute THC administration of both doses of THC induced amnesic-like effects in WT mice, and this effect was significantly reduced in 5-HT_2A_R KO animals only at the dose of 3 mg/kg (Fig 1A), suggesting that 5-HT_2A_R increases the potency of THC to induce memory impairments. Previous studies have shown that cannabinoid agonists can induce anxiogenic and anxiolytic-like responses in rodents, depending on the dose and the environmental conditions [3,7]. Low doses of cannabinoids usually induce an anxiolytic-like effect, whereas higher doses cause the opposite response. Using the previous reported dose of 0.3 mg/kg, we evaluated the involvement of 5-HT_2A_R in the anxiolytic-like effects of THC [32]. Acute administration of THC induced an anxiolytic-like effect in WT animals, while in KO mice this response was reduced (Fig 1B). In agreement, a similar acute low dose of THC increased social interaction in WT mice, whereas it decreased this response in KO animals (Fig 1C). Since anxiolytic-like behavior has been associated with changes in the activity of dorsal raphe (DR) neurons [33], we determined whether variations in DR neuronal activity induced by THC were modified in 5-HT_2A_R KO mice using extracellular electrophysiological recordings in slice preparations of DR nucleus (Fig 1D). Basal activity of DR neurons was not modified in KO with respect to WT mice (Fig 1F), indicating that 5-HT_2A_R do not exert a tonic modulation of serotonergic activity in the DR nucleus, in line with previous microdialysis data showing similar basal extracellular levels of 5-HT in the cortex of both genotypes [34]. However, KO animals were less sensitive than WT mice to the decrease in neuronal firing evoked by THC (1 nM), an effect lost at a higher THC concentration (10 nM), probably because of a ceiling effect (Fig 1E). These data suggest that specific effects of THC such as memory deficits, anxiolytic-like effects, social interaction, and DR neuronal activity are under the control of 5-HT_2A_R.

We also evaluated the role of 5-HT_2A_R in responses to chronic THC exposure. Rimonabant-precipitated THC withdrawal syndrome was evaluated in 5-HT_2A_R KO mice after chronic THC treatment. Several somatic signs of abstinence including paw tremor (Fig 1G) and sniffing (Fig 1H) were significantly attenuated in 5-HT_2A_R KO mice compared to WT animals, as well as the global withdrawal score (GWS) calculated by giving each sign a proportional weight (Fig 1I), indicating that 5-HT_2A_R is necessary for the full expression of THC withdrawal. Next, we assessed by western blot analysis whether CB_1_R levels were modified in 5-HT_2A_R KO mice following chronic treatment with THC. As a first step, we verified the specificity of the CB_1_R antibody (S1A Fig) in naïve WT and KO mice. These results show that CB_1_R are expressed at high levels in the cortex, striatum, nucleus accumbens, and hippocampus of WT mice but are virtually absent in KO mice. In animals chronically treated with THC, CB_1_R levels decreased in the hippocampus (S1B Fig) and cerebellum (S1C Fig) of both WT and KO, in agreement with previous data [35]. In the hippocampus, but not in the cerebellum, this receptor down-regulation was greater in KO mice than in WT mice treated with THC. In addition, we determined the state of the endocannabinoid system in KO mice as a control. Anandamide levels were slightly but significantly reduced (S1D Fig), while 2-arachidonoylglycerol levels were not modified in KO mice versus WT mice (S1E Fig). These findings highlight the key role played by hippocampal 5-HT_2A_R in the adaptive responses induced by chronic THC exposure.

### 5-HT_2A_R Does Not Affect the Hypolocomotor, Hypothermic, Anxiogenic, and Analgesic Effects of THC or the Reinforcing Effects of Cannabinoid Agonists

**Fig 2:**
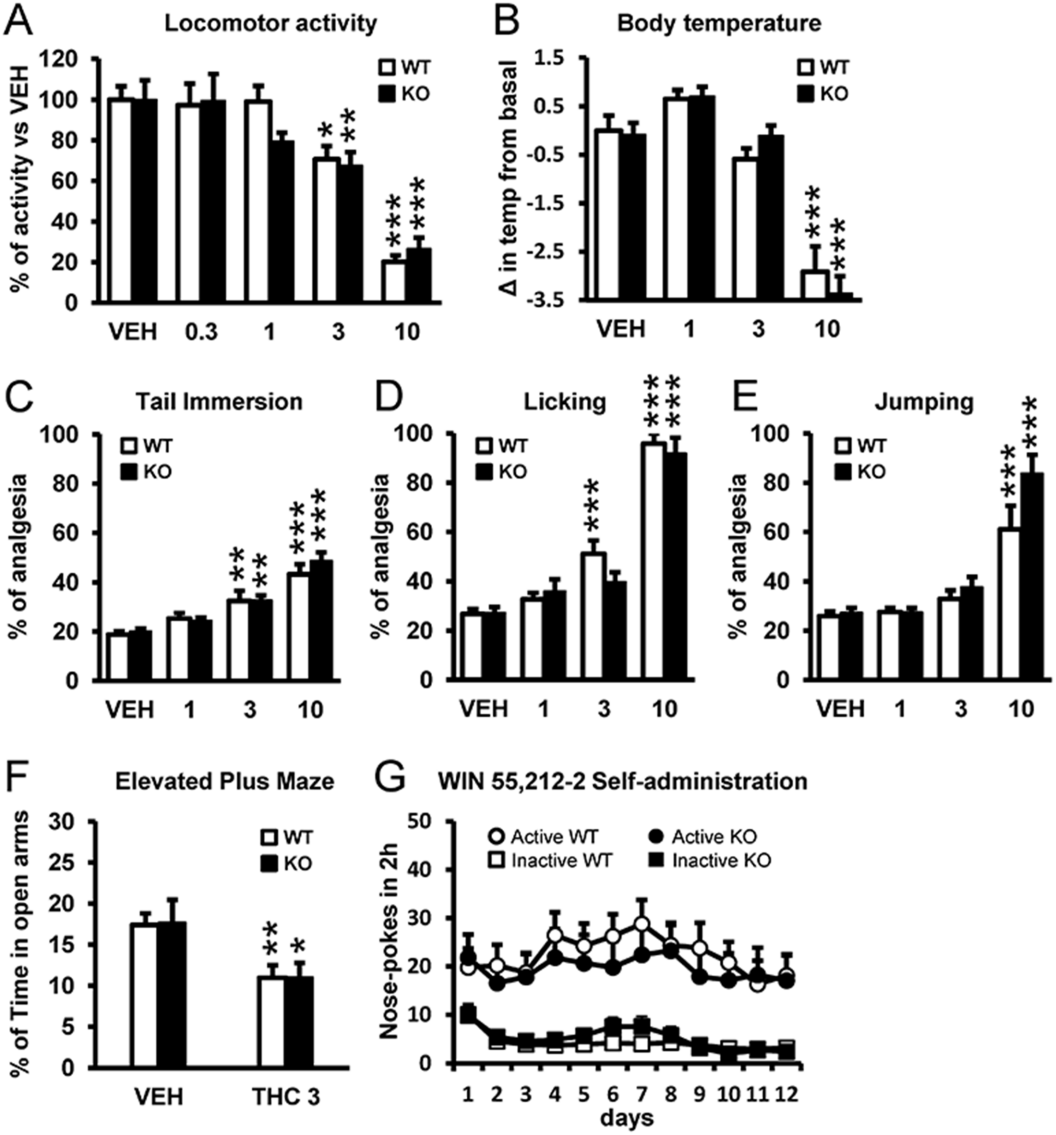
5-HT_2A_R does not mediate THC-induced hypolocomotion, hypothermia, analgesia, anxiogenic-like behavior, or the reinforcing properties of WIN 55,212–2. (A) Locomotor activity (% of baseline) was dose-dependently reduced in both WT and 5-HT_2A_R KO mice treated with THC (0.3, 1, 3, and 10 mg/kg) as compared to vehicle (VEH) administration (*n* = 5–15). (B) THC induced hypothermia at the dose of 10 mg/kg to a similar extent in WT and 5-HT_2A_R KO mice (*n* = 13–24). (C) In the tail-immersion test, percent analgesia was dose-dependently increased by THC (1, 3, and 10 mg/kg) in both WT and 5-HT_2A_R KO mice (*n* = 10–16). In the hot-plate test, the percent of analgesia as calculated from the latency to paw-licking (D) and to jumping behavior (E) was similar in WT and KO animals treated with THC (1, 3, and 10 mg/kg) as compared to VEH administration (*n* = 7–16). (F) No significant differences between genotypes were observed in anxiogenic-like behavior induced by THC (3 mg/kg) (*n* = 6–10). (G) Both WT and 5-HT_2A_R KO mice acquired WIN 55,212–2 self-administration behavior and responded equally for this drug during the 12 d of training (*n* = 12–15). Data are mean + SEM. * *p* < 0.05, ** *p* < 0.01, and *** *p* < 0.001 versus VEH-treated animals. The statistical analyses used and their corresponding F and *p*-values are shown in S1 Table.

Following the above-described differential effects in WT and 5-HT_2A_R KO animals in memory and social phenotypes, we next sought to explore other behaviors to appreciate how general the influence of 5-HT_2A_R is on THC's effects. Surprisingly, THC decreased locomotor activity in a similar dose-dependent manner in WT and KO mice (Fig 2A). Likewise, THC reduced body temperature dose-dependently and induced a profound hypothermia at 10 mg/kg in both genotypes (Fig 2B). Next, we determined the antinociceptive effects of THC in WT and KO mice using two different behavioral tests, namely, the tail-immersion and hot-plate tests. In the tail-immersion test, THC induced a comparable dose-dependent analgesic effect in WT and KO mice (Fig 2C). Similarly, the antinociception observed in the hot-plate test in terms of forepaw licking (Fig 2D) and jumping (Fig 2E) responses was comparable in WT and KO mice. To evaluate THC-induced anxiogenic-like behavior, we used the dose of 3 mg/kg, in order to avoid the locomotor suppressant effects of THC at higher doses. The acute administration of this dose induced an anxiogenic-like response in both WT and KO mice as revealed by a decrease in the percentage of time spent in the open arms of the elevated plus maze, with similar extent in both WT and KO mice (Fig 2F). Finally, the reinforcing properties of the CB_1_R agonist, WIN 55,212–2, were investigated using the intravenous operant self-administration model [36], which is the most reliable paradigm in rodents to evaluate the addictive potential of drugs of abuse [37]. Both WT and KO mice learned to discriminate between the active and inactive nose pokes and self-administered WIN 55,212–2 in a similar manner, indicating that 5-HT_2A_R does not play a role in the reinforcing properties of this cannabinoid agonist (Fig 2G), in contrast to the role played by this receptor on the reinforcing properties of psychostimulants [34]. Together, our behavioral data comparing THC's effects in WT and 5-HT_2A_R KO animals strongly point to a cross talk between CB_1_R and 5-HT_2A_R, particularly at the level of memory, anxiolytic-like behavior, social interaction, and withdrawal syndrome.

### Coexpression of 5-HT_2A_R and CB_1_R Causes Cell Signaling via Gi

**Fig 3:**
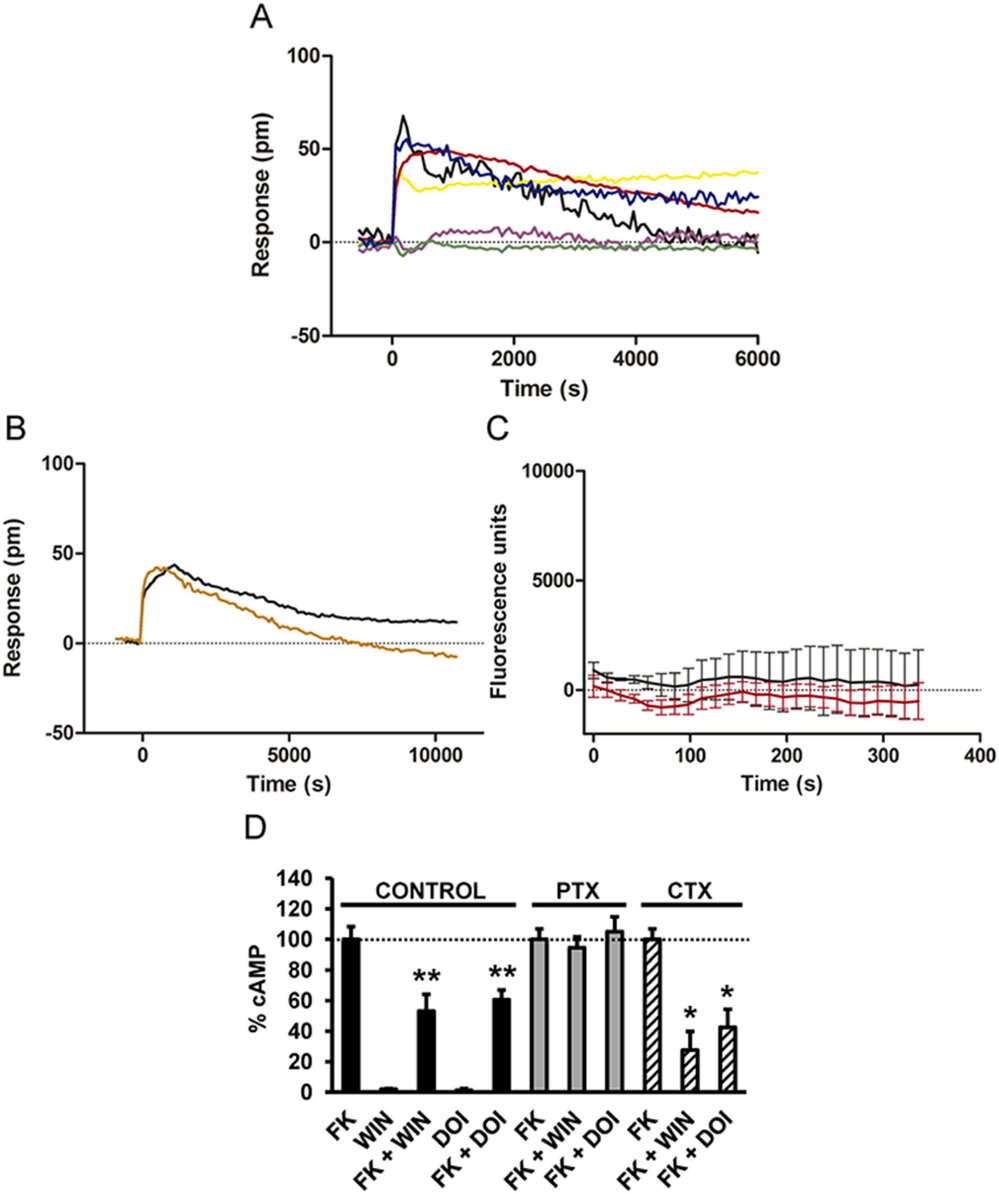
CB_1_R and 5-HT_2A_R are associated to Gi signaling when coexpressed. HEK-293Tcells expressing CB_1_R and 5-HT_2A_R receptors were used. In (A and B), the DMR analysis was performed. In (A), cells were pretreated overnight with medium (black line), with 10 ng/ml pertussis toxin (purple line), or with 100 ng/ml cholera toxin (yellow line) and were stimulated with 100 nM DOI. Alternatively, cells pretreated overnight with medium (red line), with 10 ng/ml pertussis toxin (green line), or with 100 ng/ml cholera toxin (blue line) were stimulated with 50 nM WIN 55,212–2. In (B), cells were pretreated 30 min with medium (black line) or with 1 μM of the Gq protein inhibitor YM-254890 (orange line) and were stimulated with 100 nM DOI. In all cases, the resulting picometer shifts of reflected light wavelength (pm) were monitored over time. Each curve is the mean of a representative optical trace experiment carried out in triplicates. In (C), intracellular calcium release was monitored in cells expressing CB_1_R and 5-HT_2A_R receptors treated with 100 nM DOI (black curve) or with 50 nM WIN 55,212–2 (red curve). Values are mean ± SEM of *n* = 3. In (D), cAMP production was determined in cells treated with medium (control), treated overnight with 10 ng/ml pertussis toxin (PTX), or treated 1 h with 100 ng/ml cholera toxin (CTX). Cells were stimulated with 100 nM DOI or 100 nM WIN 55,212–2 (WIN) in the absence or in the presence of 0.5 μM forskolin (FK). Values (cAMP produced in each condition minus basal stimulation in the absence of forskolin or agonists) represent mean ± SEM of *n* = 3–4 and are expressed as the percentage of the forskolin-treated cells in each conditions (120–150 pmols cAMP/10^6^ cells). One-way ANOVA followed by a Dunnett’s multiple comparison test showed a significant effect over the forskolin-alone effect in each condition (* *p* < 0.05, ** *p* < 0.01).

Next, we sought to explore at the cellular level how 5-HT_2A_R and CB_1_R might achieve the above-described cross talk between receptors. We first measured the global cellular response using dynamic mass redistribution (DMR) label-free assays, which detect changes in light diffraction in the bottom 150 nm of a cell monolayer [38]. Both CB_1_R agonists, THC and WIN 55,212–2, induced dose- and time-dependent signaling in cells only expressing CB_1_R (S2A Fig). The 5-HT_2A_R receptor agonist DOI was unable to signal, and the antagonist MDL100, 907 was unable to revert the WIN 55,212-2-induced signal in cells expressing CB_1_R (S2B Fig). The 5-HT_2A_R agonists, DOI and serotonin, induced dose- and time-dependent signaling in cells only expressing 5-HT_2A_R (S2C Fig). Furthermore, WIN 55,212–2 was unable to signal, and the antagonist rimonabant was unable to revert the DOI-induced signaling in cells expressing 5-HT_2A_R (S2D Fig), demonstrating the selectivity of the ligands. In cells stably expressing CB_1_R, WIN 55,212–2 induced a time-dependent cell signal that was inhibited by pertussis toxin (PTX), but not by cholera toxin (CTX) (S3A Fig), confirming that CB_1_R are coupled to Gi in these cells [39]. Accordingly, WIN 55,212–2 reduced the forskolin-induced cyclic adenosine monophosphate (cAMP), an effect blocked by PTX, but not by CTX or the Gq inhibitor YM-254890 (S3B Fig). In cells only expressing 5-HT_2A_R, the cell signal induced by DOI was not blocked by CTX or PTX, suggesting something other than a Gi or Gs coupling (S3C Fig). Moreover, DOI was not able to increase cAMP or decrease forskolin-induced cAMP (S3D Fig), bolstering previous studies showing that 5-HT_2A_R is coupled to Gq. In agreement, the Gq inhibitor YM-254890 completely blocked the cell response to DOI (S3E Fig). DOI was able to induce intracellular calcium release in these cells, an effect that was blocked by YM-254890 (S3F Fig), confirming that when expressed alone, 5-HT_2A_R are coupled to a Gq protein. Importantly, in cells coexpressing CB_1_R and 5-HT_2A_R, the DMR signal induced by both WIN 55,212–2 and DOI was inhibited by PTX, but not by CTX (Fig 3A). The Gq inhibitor, YM-254890, had no effect on DOI signaling (Fig 3B), and neither DOI nor WIN 55,212–2 induced intracellular calcium release in these cells (Fig 3C). These results suggest that coexpression of CB_1_R and 5-HT_2A_R causes Gi coupling. Thus, blocking of Gi is sufficient to block receptor signaling. To further support this finding, we measured changes in cAMP production upon receptor activation. In cells expressing CB_1_R and 5-HT_2A_R, both WIN 55,212–2 and DOI treatments led to a reduction in forskolin-activated cAMP production, and the effect of both ligands was sensitive to PTX, but not to CTX (Fig 3D). In addition, WIN 55,212–2 or DOI alone, in the absence of forskolin, did not modify cAMP in these cells (Fig 3D). This change of G-protein coupling of 5-HT_2A_R by coexpression of CB_1_R suggests the formation of CB_1_R-5-HT_2A_R heteromers.

### CB_1_R and 5-HT_2A_R Form Heteromers

**Fig 4:**
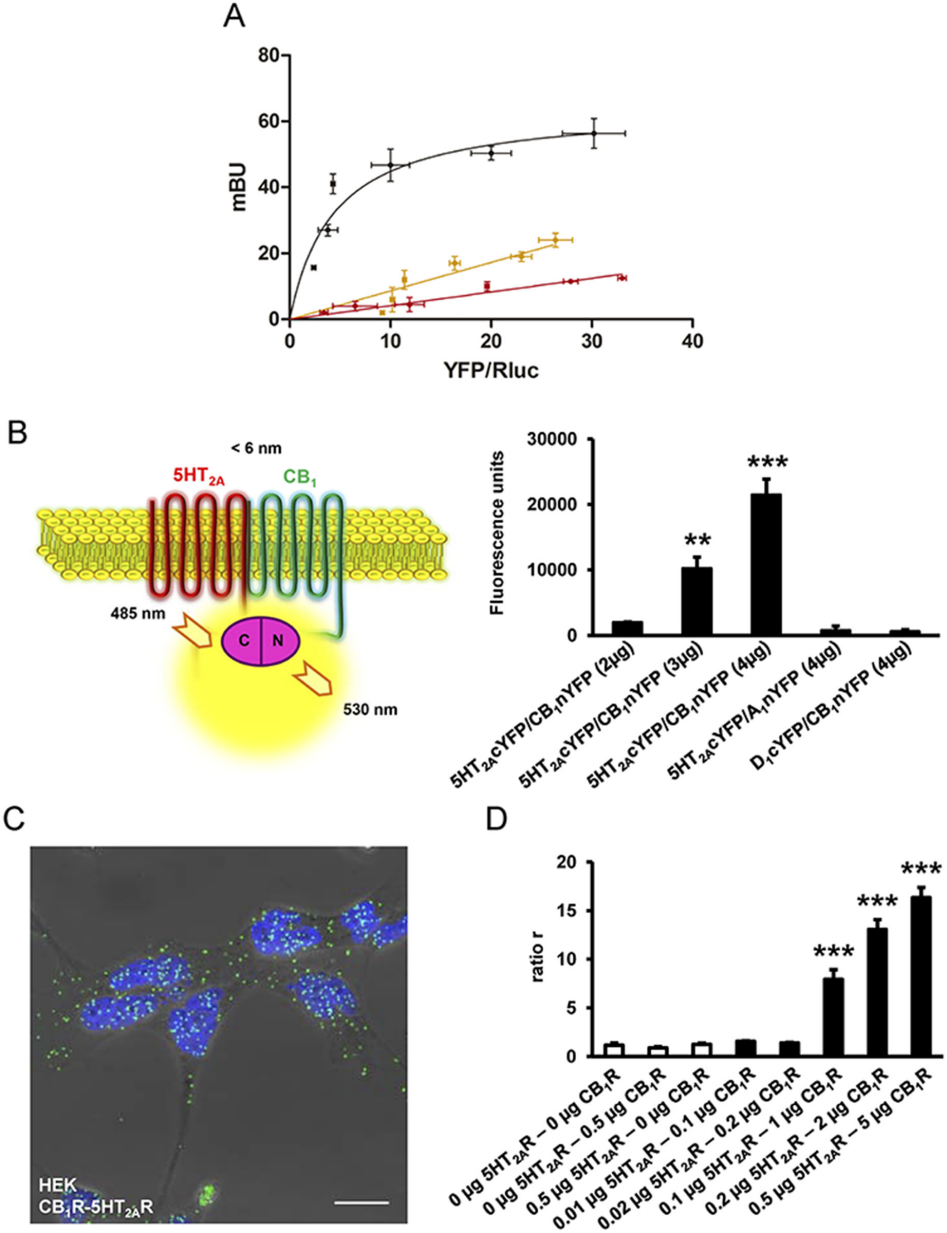
5-HT_2A_R and CB_1_R form heteromers in transfected cells. In (A), BRET saturation experiments were performed in HEK-293T cells transfected with 0.025 μg of 5-HT_2A_R-Rluc cDNA and increasing amounts of CB_1_R-YFP cDNA (0.05 μg to 1.5 μg, black curve), with 0.5 μg of dopamine D_1_R-Rluc cDNA and increasing amounts of CB_1_R-YFP cDNA (0.5 μg to 6 μg, yellow line), or with 0.025 μg of 5-HT_2A_R-Rluc cDNA and increasing amounts of adenosine A_1_R-YFP cDNA (0.05 μg to 1.5 μg, red line). The relative amount of BRET is given as a function of 100 x the ratio between the fluorescence of the acceptor (YFP) and the luciferase activity of the donor (Rluc). BRET is expressed as milli BRET units (mBU) and is given as the mean ± standard deviation (SD) of 3–6 experiments grouped as a function of the amount of BRET acceptor. In (B), a schematic representation of fluorescence complementation experiments is depicted in the left panel showing that fluorescence only appears after the YFP Venus hemiprotein complementation due to the proximity of two receptors fused to hemi-YFP Venus proteins (cYFP or nYFP). In the right panel, fluorescence at 530 nm was detected in HEK-293T cells transfected with different amounts of cDNA corresponding to both 5-HT_2A_R-cYFP and CB_1_R-nYFP (equal amount for each construct), but not in negative controls in which cells were transfected with cDNA corresponding to 5-HT_2A_R-cYFP and the noninteracting adenosine A_1_ receptor-nYFP or CB_1_R-nYFP and the noninteracting dopamine D_1_ receptor-cYFP. One-way ANOVA followed by a Dunnett’s multiple comparison test showed a significant fluorescence over basal values in HEK-293T cells (** *p* < 0.01, *** *p* < 0.001). In (C), PLAs were performed in HEK-293T cells expressing CB_1_R and 5-HT_2A_R. Confocal microscopy images (superimposed sections) are shown in which heteromers appear as green spots. In all cases, cell nuclei were stained with DAPI (blue). Scale bars = 20 μm. In (D), PLAs were performed in nontransfected HEK-293T cells, cells transiently transfected with 0.5 μg of CB_1_R or 5-HT_2A_R cDNA (negative controls, white columns), or with increasing amounts of CB_1_R and 5-HT_2A_R cDNA (black columns). In each case, the ratio between the number of green spots and the number of cells showing spots (ratio r) was calculated. One-way ANOVA followed by a Dunnett’s multiple comparison test showed a significant PLA staining over nontranfected cells (*** *p* < 0.001).

CB_1_R and 5-HT_2A_R have been traditionally considered as monomeric structural units that are coupled to intracellular heterotrimeric G-proteins. More recent evidence suggests that they can also assemble into homomers or heteromers with other G protein-coupled receptors (GPCRs) [40,41]. We hypothesized, based on the above results, that CB_1_R and 5-HT_2A_R form heteromers and their different expression in brain regions might account for the dissociation of THC behavioral responses. To test this hypothesis, we first used a bioluminescent resonance energy transfer (BRET) assay. This assay has been well established for studying GPCR interactions and has the advantage over classical immunoprecipitation approaches that it is performed in live cells over a range of protein expression levels [42,43]. A saturable BRET curve (BRET-max of 64 ± 8 milli BRET unit [mBU] and BRET_50_ of 4 ± 2) in cells expressing a constant amount of 5-HT_2A_R—renilla luciferase (Rluc) and increasing amounts of CB_1_R yellow fluorescent protein (YFP) was obtained (Fig 4A), indicating a specific interaction. Low and linear plots were observed using either dopamine D_1_R-Rluc as the donor or adenosine A_1_R-YFP as the acceptor as negative controls (Fig 4A), results consistent with nonspecific interactions [43]. These results indicate that 5-HT_2A_R and CB_1_R can form heteromers when coexpressed in cells. Further support for heteromer formation was obtained by bimolecular fluorescence complementation (BiFC) assays outlined in Fig 4B. In this assay, fluorescence only appears after correct folding of two YFP Venus hemiproteins. This occurs when two receptors fused to hemi-YFP Venus proteins (cYFP or nYFP) come within proximity. Fluorescence was detected in HEK-293T cells transfected with different amounts of cDNA corresponding to both 5-HT_2A_R-cYFP and CB_1_R-nYFP, but not in negative controls in which cells were transfected with cDNA corresponding to 5-HT_2A_R-cYFP and the noninteracting A_1_R-nYFP or CB_1_R-nYFP and the noninteracting D_1_R-cYFP (Fig 4B). Finally, in a third technique, we provided additional evidence of heteromer formation via proximity ligation assays (PLAs). This technique permits the direct detection of molecular interactions between two endogenous proteins or transfected proteins, without the need of fusion proteins. This technique is similar to immunoprecipitation but has an additional advantage of not requiring membrane solubilization. Labeling heterodimers by PLA requires both receptors to be sufficiently close to allow the two antibody-DNA probes to form double stranded segments (<17 nm), a signal that is further amplified in the presence of fluorescent nucleotides [44]. CB_1_R-5-HT_2A_R heteromers were observed as green punctate staining in HEK-293T cells coexpressing CB_1_R and 5-HT_2A_R (Fig 4C), but not in negative controls in HEK-293T cells expressing CB_1_ and D_1_ receptors or for the noninteracting CB_1_ and transferrin receptors, in spite of the expression and high colocalization of these pairs at the membrane level (S4A and S4B Fig). Also as a negative control, no PLA staining was detected in samples in which cells only expressing CB_1_R or 5-HT_2A_R alone were mixed at a 1:1 ratio (S4C Fig). Previous studies have shown PLA to be semiquantitative and particularly useful at lower expression levels [45]. To estimate the relative sensitivity of the PLA for GPCRs, experiments were performed in cells transfected with increasing cDNA amounts of 5-HT_2A_R and CB_1_R. In each case, PLA was quantified as the ratio between the number of green spots and the number of cells expressing spots (ratio r). This ratio was then represented as a function of the receptor’s cDNA transfected (Fig 4D). We observed an increase in PLA signal with increasing cDNA (Fig 4D.) These three independent approaches provide strong support for the formation of CB_1_R-5-HT_2A_R heteromers.

### Functional Characteristics of CB_1_R-5-HT_2A_R Heteromers

**Fig 5:**
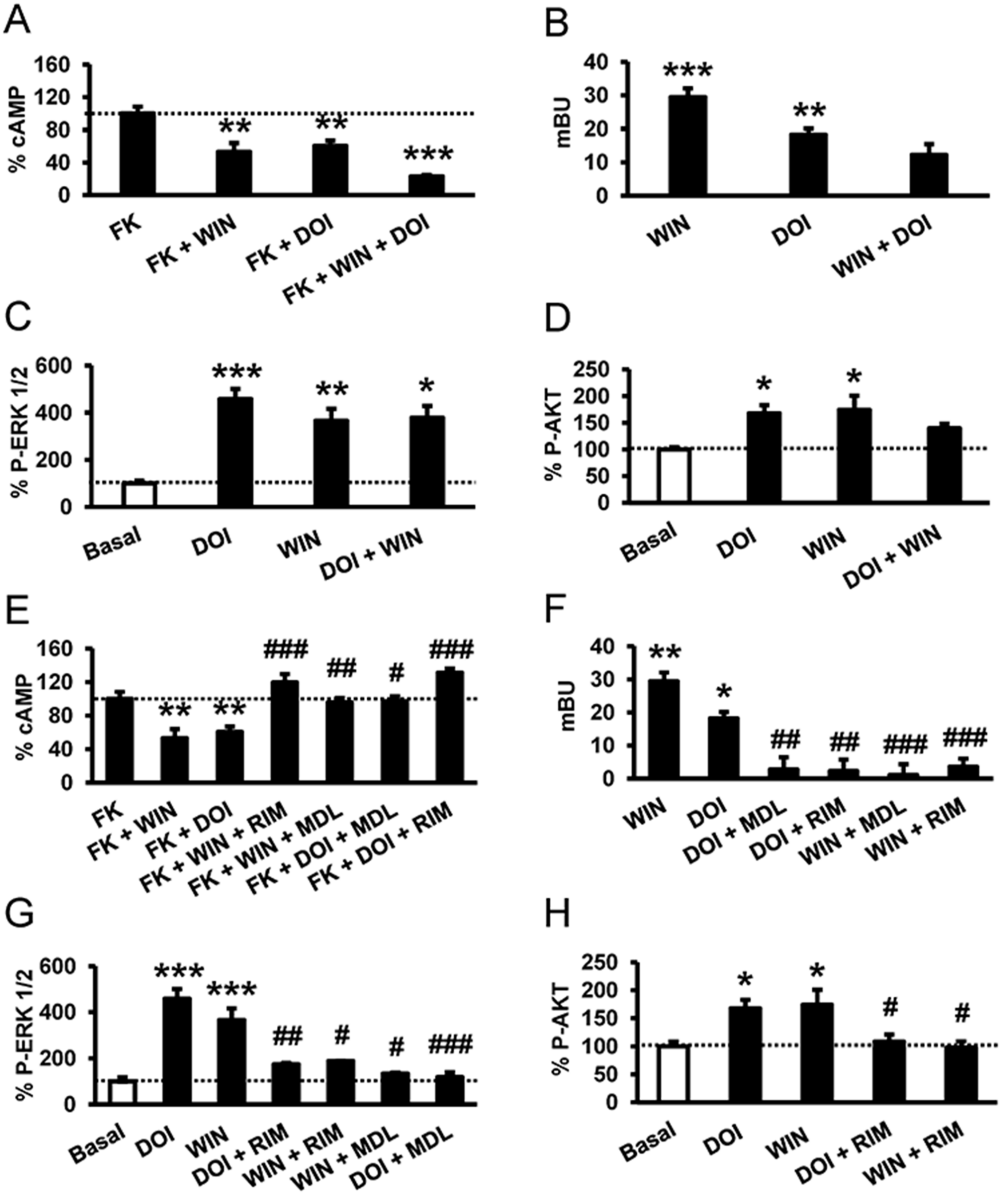
A profile of CB_1_R-5-HT_2A_R heteromer signaling. In (A and E), cAMP production was determined in HEK-293Tcells expressing CB_1_R and 5-HT_2A_R after stimulation with 100 nM DOI, 100 nM WIN 55,212–2 (WIN), or both in the absence or in the presence of 0.5 μM forskolin. In (E), cells were first preincubated either with the CB_1_R antagonist rimonabant (1 μM, RIM) or the 5-HT_2A_R antagonist MDL 100,907 (300 nM) for 20 min prior to being stimulated. Values (cAMP produced in each condition minus basal stimulation in the absence of forskolin or agonists) represent mean ± SEM of *n* = 3–4 and are expressed as the percentage of the forskolin-treated cells in each condition (120–150 pmols cAMP/10^6^ cells). One-way ANOVA followed by a Bonferroni post hoc tests showed a significant effect over the forskolin-alone effect in each condition (** *p* < 0.01, *** *p* <0.001) or of the antagonist plus agonist treatment over the agonist treatment (# *p* < 0.05, ## *p* < 0.01, ### *p* < 0.001). In (B and F), β arrestin II recruitment was measured by BRET experiments in cells transfected with 2 μg of CB_1_R cDNA, 0.2 μg of β-arrestin II-Rluc cDNA, and 1 μg of 5-H_2A_R-YFP cDNA. Cells were not preincubated with antagonist (B) or were preincubated (F) for 20 min with vehicle, rimonabant (1 μM, RIM), or MDL 100,907 (300 nM, MDL). Cells were not treated (BRET < 10) or were treated for 7 min with WIN 55,212–2 (100 nM, WIN) or DOI (100 nM) before BRET determination. Values represent mean ± SEM of *n* = 4–6. One-way ANOVA followed by a Bonferroni post hoc tests showed a significant effect over not-treated cells (* *p* < 0.05, ** *p* < 0.01, *** *p* <0.001) or of the antagonist plus agonist treatment over the agonist treatment (## *p* < 0.01, ### *p* < 0.001). In (C, D, G, and H), cells expressing CB_1_R and 5-HT_2A_R were not preincubated with antagonist (C and D) or were preincubated for 15 min with rimonabant (1 μM, RIM) or MDL 100,907 (300 nM, MDL) (G and H) and stimulated for 5 min with WIN 55,212–2 (100 nM,WIN) or DOI (100 nM). Quantification of phosphorylated ERK 1/2 (C and G) or Akt (D and H) was determined by western blot. Values, expressed as percentage of basal (nontreated cells), were mean ± SEM of *n* = 3–10. One-way ANOVA followed by a Bonferroni post hoc tests showed a significant effect over basal (* *p* < 0.05, ** *p* < 0.01, ** *p* < 0.001) or of the antagonist plus agonist treatment over the agonist treatment (# *p* < 0.05, ## *p* < 0.01, ### *p* < 0.001).

A common consequence of heteromer formation is altered downstream signaling upon dual stimulation of the receptors in the heteromer [46–48]. To examine whether this may be the case for CB_1_R-5-HT_2A_R- heteromers, we determined signaling through adenylate cyclase, arrestin recruitment, the ERK 1/2 pathway, and the Akt pathway in cells expressing both receptors. In cells stimulated with forskolin and treated with WIN 55,212–2, DOI, or both, we found that costimulation led to reduced cAMP production (Fig 5A). To examine whether costimulation led to changes in β-arrestin II recruitment compared to single stimulation, the agonist-induced interaction of arrestin with the receptors was measured by BRET in cells expressing β-arrestin II-Rluc, 5-HT_2A_R-YFP, and CB_1_R. Both agonists, DOI and WIN 55,212–2, recruited β-arrestin II, but costimulation led to a significant decrease in arrestin recruitment (Fig 5B), indicating that costimulation reduces cell signaling. Both CB_1_R and 5-HT_2A_R agonists induced the activation of ERK 1/2 and Akt pathways in a time- and dose-dependent manner (S5 Fig). Measuring ERK 1/2 phosphorylation (Fig 5C) or Akt phosphorylation (Fig 5D), the costimulation with WIN 55,212–2 and DOI, surprisingly, did not increase the phosphorylation levels reached by each agonist separately. This response was not due to a change in the optimum time response for ERK 1/2 (S6A Fig) or Akt phosphorylation (S6B Fig). Taken together, these data suggest that costimulation of CB_1_R-5-HT_2A_R heteromers leads to reduced cell signaling. Some GPCR heteromers have been found to display cross antagonism, the ability of an antagonist of one receptor to antagonize the signaling of the partner receptor [49,50]. Cross antagonism requires direct protein—protein interaction since antagonists do not signal on their own. When cells coexpressing both receptors were pretreated with the CB_1_R antagonist rimonabant and then stimulated with the CB_1_R agonist WIN 55,212–2 or the 5-HT_2A_R agonist DOI, surprisingly no decreases in cAMP (Fig 5E), no β-arrestin II recruitment (Fig 5F), and no phospho-ERK 1/2 (Fig 5G) or phospho-Akt (Fig 5H) were observed. These results indicate that rimonabant blocks both CB_1_R and 5-HT_2A_R signaling. Analogously, the signaling in cAMP (Fig 5E), β-arrestin II recruitment (Fig 5F), and phospho-ERK 1/2 (Fig 5G) or phospho-Akt (Fig 5H) induced by both the CB_1_R agonist WIN 55,212–2 and the 5-HT_2A_R agonist DOI were completely blocked when cells were pretreated with the 5-HT_2A_R antagonist MDL 100,907. This cross antagonism is not due to the lack of specificity of the ligands since the 5-HT_2A_R agonist DOI and the antagonist MDL100,907 were unable to signal or modify the CB_1_R signaling in cells only expressing CB_1_R (S6C Fig). Furthermore, the CB_1_R agonist WIN 55,212–2 and the antagonist rimonabant did not modify the 5-HT_2A_R signaling in cells only expressing 5-HT_2A_R (S6D Fig). In total, these results demonstrate that CB_1_R-5-HT_2A_R heteromers display bidirectional cross antagonism.

### Molecular Basis of Cross Antagonism in CB_1_R-5-HT_2A_R Heteromers

**Fig 6:**
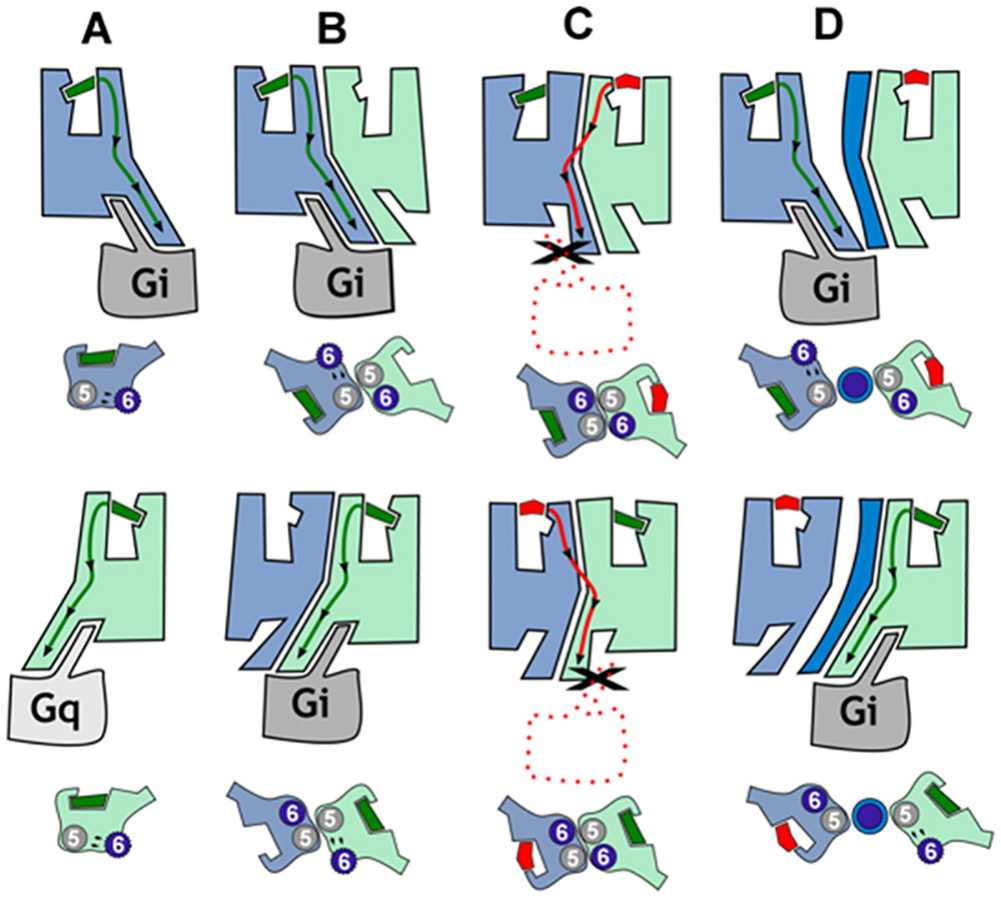
Proposed functional properties of CB_1_R-5-HT_2A_R heteromers. In (A), agonist binding to CB_1_R (blue) or 5-HT_2A_R (light green) triggers the conformational changes of TMs 5 and 6, opening the intracellular cavity for Gi and Gq binding, respectively. In (B), the formation of the CB_1_R-5-HT_2A_R heteromer makes both receptors signal via Gi. In (C), rimonabant binding to CB_1_R or MDL 100,907 to 5-HT_2A_R stabilizes the closed conformation of the receptor, facilitating heterodimerization via TMs 5 and 6 as in the crystal structure of the μ-opioid receptor. In this assembly, both protomers are locked in the closed conformation since the opening of TMs 5 and 6 for G-protein binding is not feasible (see S7 Fig). Bidirectional cross antagonism is due to the fact that antagonist binding to any protomer must, in addition to its common role in a monomeric signaling unit, disrupt this very stable four-helix association. (D) In agreement, bidirectional cross antagonism is abrogated following treatment with TM 5 or TM 6 interference peptides (dark blue), which disrupt the heteromer structure.

**Fig 7:**
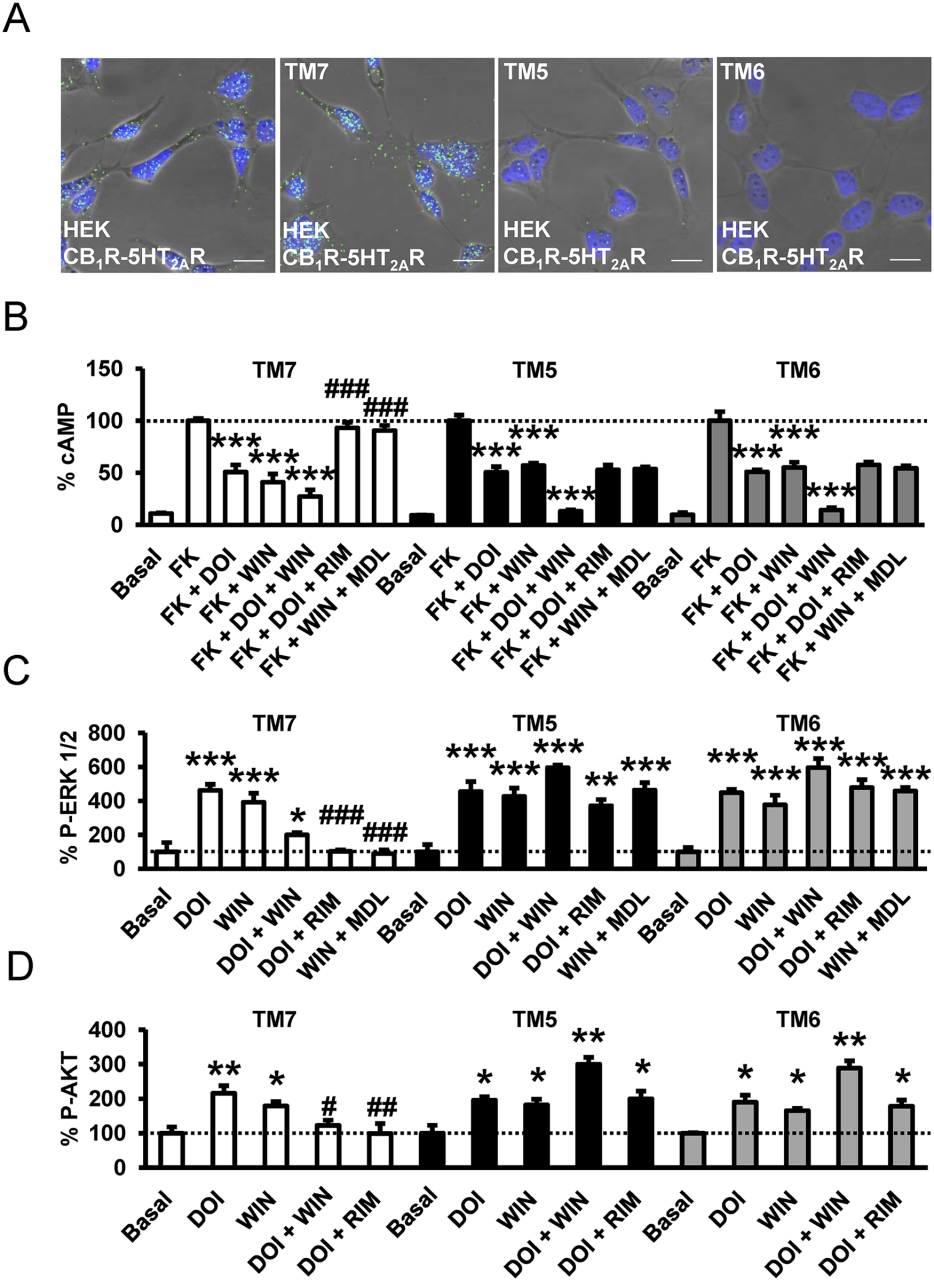
Interacting protomer domains in CB_1_R-5-HT_2A_R heteromers and heteromer disruption by TM interference peptides. In (A), HEK-293T cells expressing CB_1_R and 5-HT_2A_R were treated for 4 h with vehicle (left panel) or 4 μM of CB_1_R TM 7, TM 5, or TM 6 interference peptides before performing proximity ligation assays. Confocal microscopy images (superimposed sections) are shown in which heteromers appear as green spots in cells treated with vehicle and with TM 7 interference peptide, but not in cells treated with TM 5 or TM 6 interference peptides. In all cases, cell nuclei were stained with DAPI (blue). Scale bars = 20 μm. In (B–D), HEK-293T cells expressing CB_1_R and 5-HT_2A_R were preincubated for 20 min with rimonabant (1 μM, RIM) or MDL 100,907 (300 nM, MDL) before stimulation for 10 min (B) or 5 min (C, D) with the CB_1_R agonist WIN 55,212–2 (100 nM), the 5-HT_2A_R agonist DOI (100 nM), or both in the presence (B) or absence (C, D) of 0.5 μM forskolin. In (B), cAMP production was determined. Values represent mean ± SEM of *n* = 3–9 and are expressed as the percentage of the cAMP produced in forskolin-treated cells. Quantification of phosphorylated ERK 1/2 (C) or Akt (D) was determined by western blot. Values, expressed as a percentage of basal (nontreated cells), were mean ± SEM of *n* = 3–6. One-way ANOVA followed by a Bonferroni post hoc tests showed a significant effect over forskolin’s effects alone in each condition (B) or over basal (C, D) (* *p* < 0.05, ** *p* < 0.01, *** *p* <0.001) or of the antagonist plus agonist treatment over the agonist treatment (# *p* < 0.05, ## *p* < 0.01, ### *p* < 0.001).

To understand how receptor—receptor interactions might facilitate the above-mentioned cross antagonism and to potentially design a biochemical tool to disrupt these interactions, we took advantage of the exponential growth in the number of solved GPCR structures, in the form of monomers or homo-oligomers, bound to either agonists, antagonists, inverse agonists, or in complex with the G-protein to model heteromer activation (S7 Fig) [51]. Agonist binding at the extracellular side triggers small local structural changes near the binding site [52] that are translated into larger-scale helix movements at the intracellular site [53]. Specifically, agonists increase signaling by opening an intracellular cavity, required for the binding of the C-terminal α5 helix of the G-protein, through the movement of TM 5 and TM 6. Conversely, inverse agonists decrease the basal, agonist-independent level of signaling by closing this cavity. Our findings that CB_1_R-5-HT_2A_R heteromers display bidirectional cross antagonism led us to suggest that the antagonist-bound conformation of protomer A allosterically prevents the opening of the intracellular cavity of protomer B. To our knowledge, the molecular basis of this bidirectional cross antagonism has not been described. Recently, the crystal structure of the μ-opioid receptor has shown a novel mode of receptor dimerization via TMs 5 and 6 [54]. In this assembly, TMs 5 and 6 of protomer A form a very stable four-helix bundle with TMs 5 and 6 of protomer B (S7A Fig). This high surface complementarity in the heteromer, within the four-helix bundle interface, prevents the opening of the intracellular cavity (S7A Fig). Thus, we hypothesized that bidirectional cross antagonism in the CB_1_R-5-HT_2A_R heteromer is due to antagonist binding to either protomer that stabilizes the inactive conformation of TM 5 and TM 6 and to the subsequent formation of this very stable four-helix association. As a consequence, the action of agonists is blocked at both receptors (see Fig 6A–6D for details). To test this hypothesis, we investigated if synthetic peptides with the sequence of TMs 5, 6, and 7 (as negative control) of CB_1_R, fused to HIV TAT, were able to disrupt receptor heterodimerization and the observed bidirectional cross antagonism. This approach has been used by us and others previously [55,56]. We first checked by immunocytochemistry that TM 5, TM 6, and TM 7 interference peptides do not appreciably change the expression and colocalization of CB_1_R and 5-HT_2A_R at the membrane level (S8 Fig). We found that pretreatment with TM 5 and TM 6 (but not TM 7) interference peptides of cells expressing CB_1_R-nYFP and 5-HT_2A_R-cYFP disrupt the heteromer structure as revealed by a loss of fluorescence in BiFC assays (S9 Fig). These results were further confirmed by PLA assays. CB_1_R-5-HT_2A_R heteromers were observed as green punctate staining in HEK-293T cells not treated or treated with the TM 7 interference peptide, but they were absent in cells treated with TM 5 or TM 6 interference peptides (Fig 7A). Notably, the cross antagonism was not observed at the level of cAMP (Fig 7B), ERK 1/2 phosphorylation (Fig 7C), and Akt phosphorylation (Fig 7D) in HEK-293T cells expressing CB_1_R and 5-HT_2A_R and treated with TM 5 or 6 peptides. The effect of the peptides was specific to the heteromer, as single activation of the individual receptors still led to signaling in the presence of the peptides. Importantly, these results indicate that negative cross talk and cross antagonism require receptor—receptor interaction and are specific biochemical characteristics of the heteromer that can be used as a fingerprint to detect the heteromer [47,49,57]. These results suggest that receptor heterodimerization occurs via TM 5 and TM 6, which facilitates the observed cross antagonism. GPCRs are dynamic proteins that permit rapid small-scale structural fluctuations and pass through an energy landscape to adopt a number of conformations ranging from inactive to active. Our results have shown that in the case of receptor heterodimerization via TM 5 and TM 6, one of the protomers allosterically modulates the functional properties (energy landscape) of the interacting receptor (S7B Fig).

### Differential Expression of Functional CB_1_R-5-HT_2A_R Heteromers in the Brain

**Fig 8:**
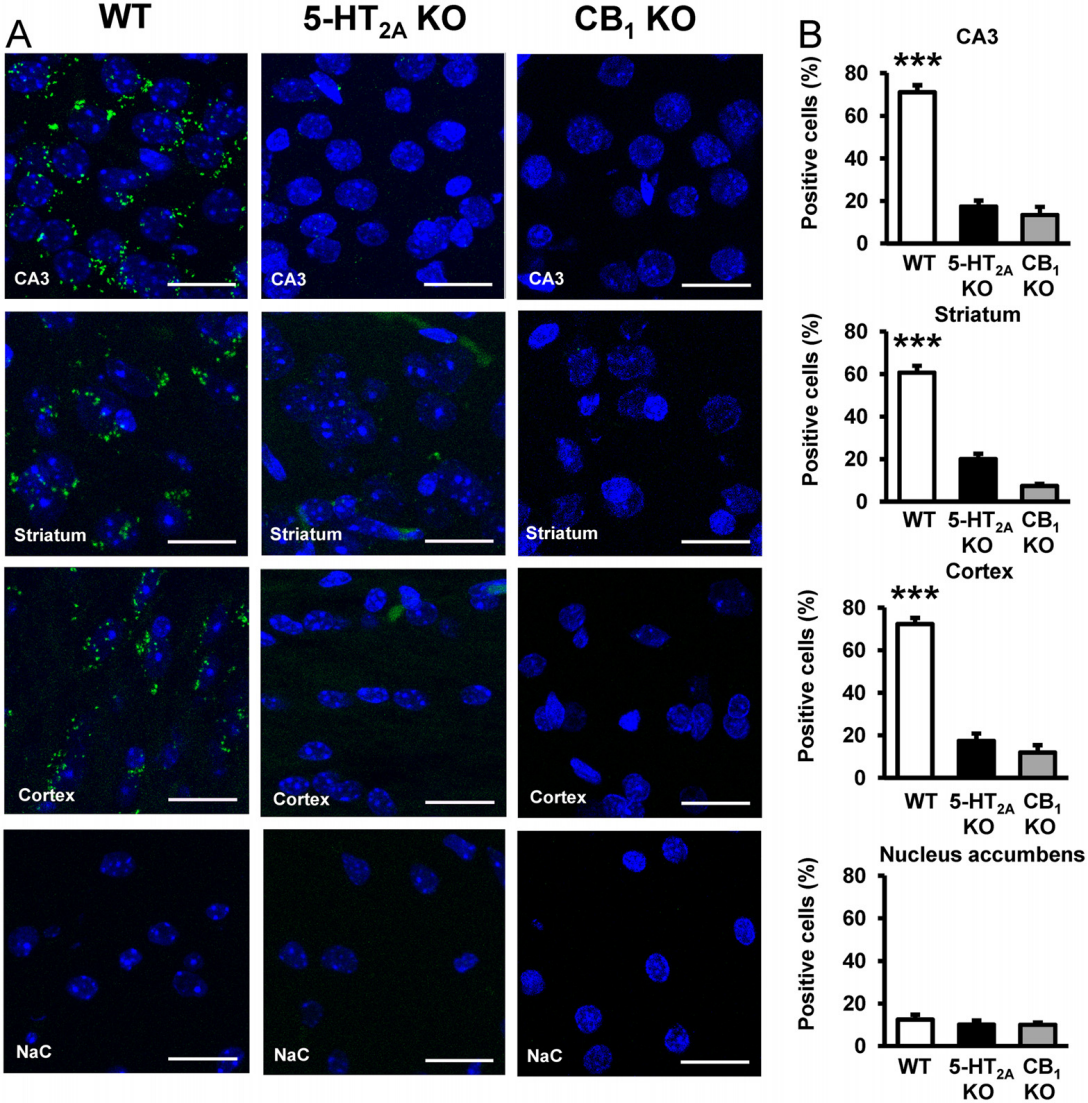
Differential expression of CB_1_R-5-HT_2A_R heteromers in the brain detected by in situ PLAs. In (A), PLAs were performed using slices of mouse hippocampus CA3, caudate-putamen (striatum), cortex (somatomotor layers 1, 2, and 3) or nucleus accumbens (NaC). Confocal microscopy images (superimposed sections) are shown in which heteromers appear as green spots in WT mice, but not in 5-HT_2A_R KO or CB_1_R KO mice in the hippocampus, caudate-putamen, and cortex. Any staining was observed in the nucleus accumbens of either WT or KO animals. In all cases, cell nuclei were stained with DAPI (blue). Scale bars = 20 μm. In (B), the number of cells containing one or more green spots is expressed as the percentage of the total number of cells (blue nucleus) in the hippocampus, striatum, cortex, and nucleus accumbens of WT (white bars), 5-HT_2A_R KO (black bars), or CB_1_R KO (grey bars) mice. Data (percentage of positive cells) are the mean ± SEM of counts in 4–9 different fields (see experimental procedures). Student’s *t* test showed a significant effect over 5-HT_2A_R KO or over CB_1_R KO mice in each condition (*** *p* < 0.001).

**Fig 9:**
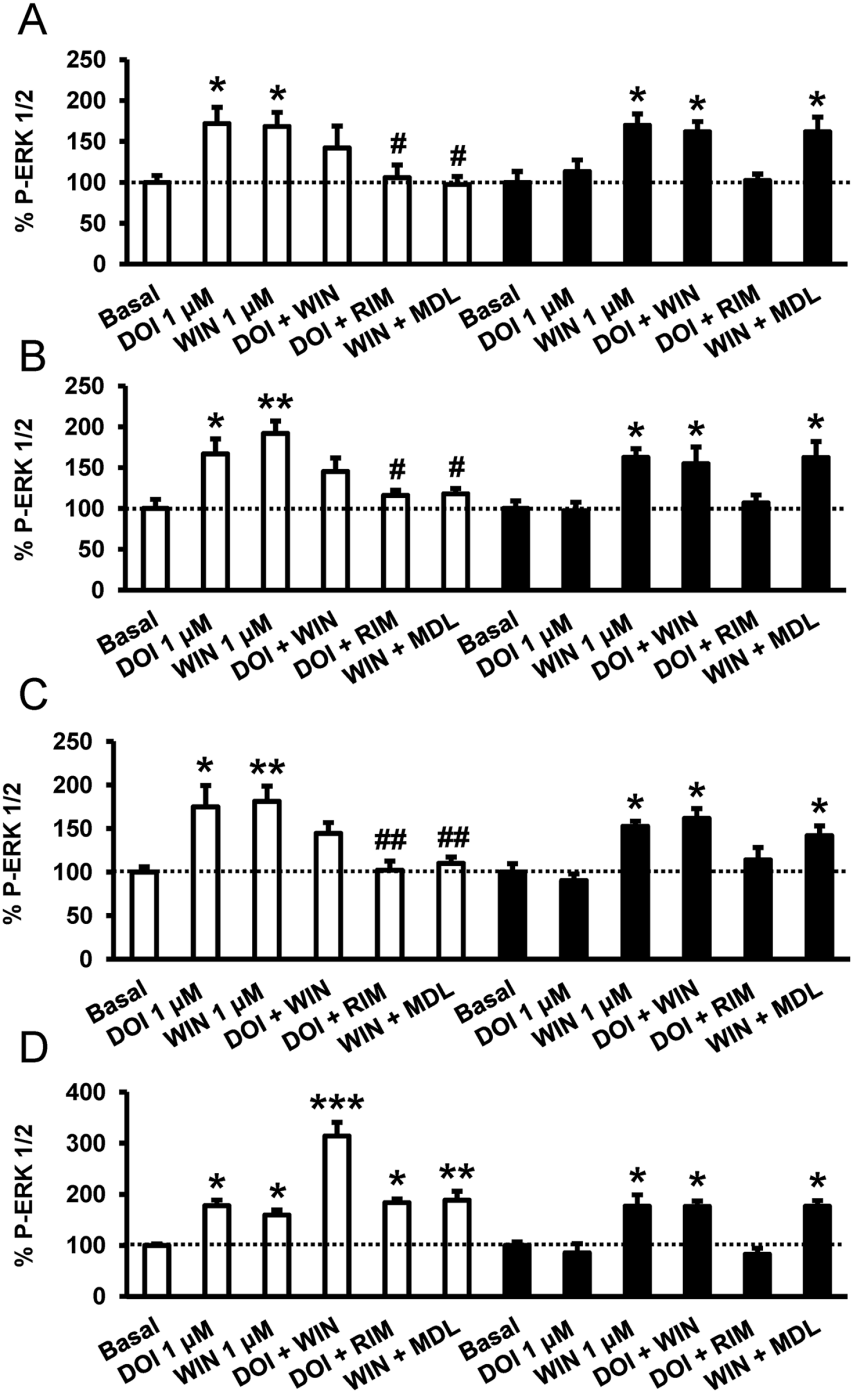
Differential expression of CB_1_R-5-HT_2A_R heteromers in the brain detected by heteromer signaling. Slices from the hippocampus (A), caudate-putamen (B), cortex (C), and nucleus accumbens (D) of WT mice (white bars) and 5-HT_2A_R KO mice (black bars) were preincubated or not with CB_1_R antagonist rimonabant (1 μM, RIM) or the 5-HT_2A_R antagonist MDL 100,907 (300 nM, MDL) for 20 min before the addition of the CB_1_R agonist WIN 55,212–2 (1 μM, WIN), the 5-HT_2A_R agonist DOI (1 μM), or both for an additional incubation period of 10 min. ERK 1/2 phosphorylation was determined by western blot. Immunoreactive bands from three to seven slices obtained from ten WT or KO animals were quantified for each condition. Values represent mean ± SEM of the percentage of phosphorylation relative to basal levels found in untreated slices. No significant differences were obtained between the basal levels of the WT and the KO mice. One-way ANOVA followed by Bonferroni post hoc tests showed a significant (* *p* < 0.05, ** *p* < 0.01, *** *p* < 0.001) effect over basal or of the antagonist plus agonist treatment over the agonist treatment (# *p* < 0.05, ## *p* < 0.01).

CB_1_R-5-HT_2A_R heteromer expression in tissue was analyzed by in situ PLA using specific primary antibodies directed against CB_1_R and 5-HT_2A_R that have been validated in WT, CB_1_R KO, and 5-HT_2A_R KO mice (Fig 8) [58]. CB_1_R-5-HT_2A_R heteromers were observed as punctate green spots in cells with DAPI-stained nuclei in slices from hippocampus (CA3 region), dorsal striatum (caudate-putamen), and cortex (somatomotor layers 1, 2, and 3), all areas where both receptors are expressed [13], of WT animals, but not in 5-HT_2A_R or CB_1_R KO animals (Fig 8A) or in negative controls (S10 Fig). In all cases, staining was observed in a relatively high percentage of cells (60%–70%) (Fig 8B). Interestingly, no green spots were detected in slices from nucleus accumbens in either WT or KO animals, indicating a differential expression of heteromers in the brain (Fig 8). To further support the existence of heteromer expression in these brain regions, we used the heteromer specific biochemical characteristics identified above (negative cross talk and cross antagonism) as a heteromer fingerprint to detect heteromers in situ. We measured ERK 1/2 phosphorylation in isolated brain slices from the hippocampus (Fig 9A), dorsal striatum (Fig 9B), cortex (Fig 9C), or nucleus accumbens (Fig 9D) of WT mice. As expected when slices were treated with WIN 55,212–2 or DOI, ERK 1/2 phosphorylation was induced. As in cells, hippocampal, dorsal striatal, and cortical cell signaling was not increased when slices were coactivated with both agonists, again suggesting reduced signaling (Fig 9A–9C, white bars). In addition, p-ERK 1/2 levels induced after treatment with DOI were lowered when the slices were pretreated with the CB_1_R antagonist rimonabant, while the 5-HT_2A_R antagonist MDL 100,907 blocked the activation induced by WIN 55,212–2 (Fig 9A–9C, white bars). This cross antagonism mirrors what was observed in transfected cells and serves as biochemical evidence that heteromers are both expressed and functional. Importantly, nucleus accumbens from WT mice showed increased signaling upon dual stimulation and no cross antagonism (Fig 9D, white bars), supporting the lack of expression of the heteromer as previously observed by the lack of PLA staining. To confirm that the results were indeed due to the expression of heteromers, we repeated the experiments in 5-HT_2A_R KO mice. No cross talk or cross antagonism was observed in slices from the cortex, striatum, and hippocampus of KO mice (Fig 9, black bars). The lack of heteromerization in the nucleus accumbens is not due to a lack of receptor expression in this tissue since both agonists DOI and WIN 55,212–2 induced a signal very similar to the one induced in the other brain regions, where the heteromer fingerprint or PLA staining was observed (Fig 9A–9D). The above results demonstrate the differential expression of functional CB_1_R-5-HT_2A_R heteromers in brain tissue.

### CB_1_R-5-HT_2A_R Heteromers Are Involved in the Amnesic and Anxiolytic-like Behavior Induced by THC

**Fig 10:**
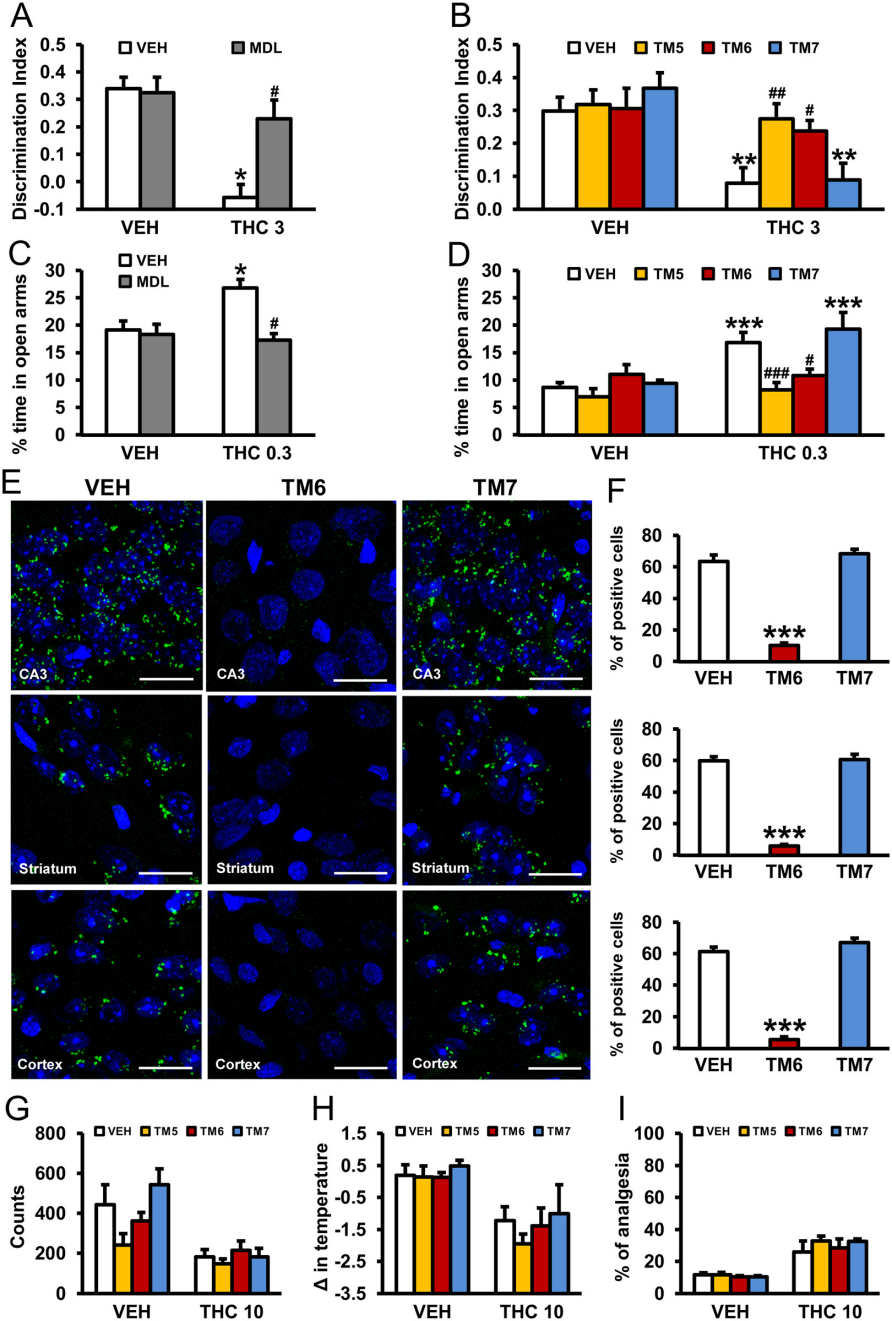
Prevention of THC-induced amnesic and anxiolytic-like effects by pharmacological blockade of 5-HT_2A_R or by CB_1_R-5-HT_2A_R heteromer disruption with TM interference peptides. The amnesic effects of THC (3 mg/kg) observed in C57BL/6J mice in the novel object recognition test were abrogated by pretreatment with the 5-HT_2A_R antagonist, MDL 100,907 (0.01 mg/kg) (A) and by pretreatment with TM 5 and TM 6, but not TM 7, interference peptides (0.2 μg/ 2μl ICV) (B) (*n* = 5–9). The anxiolytic effects of THC (0.3 mg/kg) observed in the elevated plus maze in C57BL/6J mice were blocked by pretreatment with the 5-HT_2A_R antagonist, MDL 100,907 (0.01 mg/kg) (C) and by pretreatment with TM 5 and TM 6, but not TM 7, interference peptides (0.2 μg/ 2μl ICV) (D) (*n* = 4–11). The data represent mean + SEM. * *p* < 0.05, ** *p* < 0.01, *** *p* < 0.001 versus vehicle, # *p* < 0.05 versus THC-treated mice. In (E), PLA performed in hippocampal CA3, striatal (caudate-putamen), and cortical (somatomotor layers 1, 2, and 3) slices from mice treated with VEH, TM 6, and TM 7 interference peptides (0.2 μg/ 2μl ICV). Confocal microscopy images (superimposed sections) are shown in which heteromers appear as green spots in VEH and TM 7-treated mice, but not in mice treated with TM 6 interference peptides. In all cases, cell nuclei were stained with DAPI (blue). Scale bars = 20 μm. In (F), the number of cells containing one or more green spots is expressed as the percentage of the total number of cells (blue nucleus) in the hippocampus, striatum, and cortex (top to bottom). Data (percentage of positive cells) are the mean ± SEM of counts in 8–12 different fields. *** *p* < 0.001 versus vehicle-treated mice. Pretreatment with TM 5, TM 6, or TM 7 peptides (0.2 μg/ 2μl ICV) had no significant effects on hypolocomotion (G), hypothermia (H), or analgesia (I) induced by THC (10 mg/kg) in C57BL/6J mice. The statistical analyses used and their corresponding F and *p*-values are shown in S1 Table.

In order to implicate the involvement of the heteromer in the behavioral effects of THC in vivo, we evaluated cross antagonism in WT mice. Thus, the effects of the 5-HT_2A_R antagonist MDL 100,907 on THC-induced memory impairments using the object recognition test and on its anxiolytic-like properties using the elevated plus maze were evaluated. THC (3 mg/kg) induced significant memory impairments in vehicle-treated animals, but not in mice pretreated with MDL 100,907 (0.01 mg/kg) (Fig 10A). We further confirmed that this effect was mediated by the heteromer since THC-induced memory impairments were not observed in WT animals previously treated with TM5 or TM6 peptides (0.2 μg/2 μl ICV) but were present in animals receiving the TM7 peptide (Fig 10B). Similarly, THC-induced anxiolytic-like effects were prevented by MDL 100,907 administration (Fig 10C) and by ICV infusion of TM5 and TM6 peptides, but not by TM7 (Fig 10D). Importantly, using PLA we were able to demonstrate that administration of TM5 and TM6, but not TM7, peptides was able to disrupt the heteromer in vivo. In hippocampal CA3, striatal (caudate-putamen) or cortical (somatomotor layers 1, 2, and 3) slices from mice treated with vehicle or TM7 peptide (0.2 μg/2μl ICV) heteromers appear as green spots, a staining not seen in mice treated with equivalent amounts of TM6 peptide (Fig 10E and 10F). These results demonstrate that CB_1_R-5-HT_2A_R heteromers are involved in the amnesic and anxiolytic-like behavior induced by THC.

In support of the involvement of 5-HT_2A_R in only certain effects of THC, preadministration (0.2 μg/2 μl ICV) of TM 5, TM 6, or TM 7 interference peptides did not change THC-induced hypolocomotion (Fig 10G), hypothermia (Fig 10H), or analgesia (Fig 10I). This differential effect was also observed when 5-HT2_A_R KO mice and WT littermates were compared (S11 Fig). Preadministration of TM 6, but not TM 7, peptides blocked the THC-induced changes in the discrimination index and percent of time in open arms in WT mice (S11A and S11B Fig), but neither TM 6 nor TM 7 altered the lack of effect of THC in KO mice (S11F and S11G Fig). In both WT (S11C–S11E Fig) and 5-HT_2A_R KO (S11H–S11J Fig) mice, the effect of THC on locomotion, body temperature, and analgesia was not altered by preadministration of TM 6 or TM 7 peptides. These findings provide evidence for the in vivo requirement of the CB_1_R-5-HT_2A_R heteromer to be intact in order to observe the amnesic and anxiolytic-like behavior induced by THC but not in other THC-mediated effects.

## Discussion

While exploring the neurobiological mechanisms underlying THC-induced cognitive impairment, we discovered an unexpected role of the 5-HT_2A_R. Our findings lead to three major conclusions. First, behavioral studies carried out in mice lacking 5-HT_2A_R revealed a remarkable 5-HT_2A_R-dependent dissociation in the beneficial antinociceptive effects of THC and its detrimental amnesic properties. Second, CB_1_R and 5-HT_2A_R form heteromers that are expressed and functionally active in specific brain regions involved in memory impairment. Third, to observe the negative cognitive effects of THC, these receptors must be functionally interacting, as administration of a 5-HT_2A_R antagonist or selective disruption of the CB_1_R-5-HT_2A_R heteromers by ICV infusion of synthetic interference peptides in WT mice abrogated the memory deficits induced by THC and its anxiolytic-like effects, but not its antinociceptive properties.

Previous studies have suggested interactions between endocannabinoids and 5-HT, although the extent of this alleged reciprocal interaction and the molecular mechanisms involved have been difficult to ascertain. Here we found that the amnesic, anxiolytic, and pro-social-like effects induced by THC, as well as the manifestations of THC withdrawal syndrome, were reduced in mice with constitutive deletions of 5-HT_2A_R. In contrast, 5-HT_2A_R deletion did not modulate the acute hypolocomotor, hypothermic, anxiogenic, and antinociceptive effects of THC or the reinforcing effects of the cannabinoid agonist, WIN 55,212–2. These data demonstrate for the first time, to our knowledge, that 5-HT_2A_R modulates specific behavioral responses related to CB_1_R activation by THC. There seemed to be three plausible explanations for the differential effects of THC and its dependence on 5-HT_2A_R: (1) interactions only at the level of circuitry, (2) circumstantial cross talk at the level of intracellular signaling, or (3) direct protein—protein interaction that can modify receptor function. Although our study cannot completely count out interactions at the level of circuitry, we have clearly observed cross talk in transfected cells that would circumvent the need for circuitry connections. Indeed, both receptors are coexpressed in the hippocampus, where they participate in memory processing [16,17,59]. They are also colocalized in the cerebral cortex, hypothalamus, striatum, and nucleus accumbens, brain areas implicated in reward processing and affective disorders, including anxiety [60–63]. To delineate between circumstantial intracellular cross talk and direct protein—protein interaction, we tested whether the receptors could form complexes if coexpressed, which we found to be the case. We then were able to show that the signaling cross talk observed required this receptor—receptor interaction both in vitro and in vivo. The dissociation observed regarding the involvement of this heteromeric complex in the memory impairments and the antinociception observed following THC administration were corroborated in our in vivo studies. When the heteromer was disrupted by ICV infusion of interference peptides TM5 and TM6, we observed blunted amnesic and anxiolytic, but not antinociceptive, effects of THC selectively in WT mice. Interference peptides have been successfully used in earlier studies to ascertain the role of heterodimer formation in physiological functions [56] and in behavioral models of mood disorders [64]. It appears then that serendipitous signaling cross talk is not a sufficient explanation for the dependence on 5-HT_2A_R for THC’s effects. A more plausible explanation might be that these receptors present different degrees of interaction depending on the cell type or cell location. Such a scenario would predict the existence of different populations of CB_1_R. One population when stimulated with THC provides a certain level of cellular signaling that impacts on neurons influencing locomotion or antinociceptive effects, while a separate population of CB_1_R coupled to 5-HT_2A_R would provide altered cell signaling upon exposure to THC that directly influences memory or anxiolytic-like effects. In agreement, CB_1_R in glutamatergic cells have been recently reported to have a much higher coupling to G-proteins than CB_1_R in GABAergic cells, sustaining the possibility of different functional populations of the receptor [65]. Support for this idea is provided by our results showing that 5-HT_2A_R and CB_1_R form heteromers in specific brain structures, such as the cortex, hippocampus, and striatum, but not in the nucleus accumbens, a key structure of the reward circuit. Several drugs of abuse, including cannabis, increase dopamine release in the nucleus accumbens [66], and an interaction between CB_1_R and dopamine D_2_ receptor signaling has been suggested in this area [67]. Our results showing no heteromer formation in the nucleus accumbens, together with the finding that 5-HT_2A_R does not modulate cannabinoid (WIN 55,212–2) reinforcing properties, suggest that these receptors are not involved in the modulation of dopamine responses in this structure. Moreover, our data showing a reduction in p-ERK 1/2 via the heteromer are particularly interesting since ERK signaling is important for long-term synaptic plasticity [68], which plays a crucial role in learning and memory.

Although we are unable to speculate on the amount of heteromers in the different regions, it is clear that the lack of heteromers in the nucleus accumbens is not due to lack of expression, as both receptors were still able to signal upon receptor stimulation at levels equal to the other brain regions. These data suggest that heteromer formation is not simply due to overexpression of the receptors in these regions and that there exists a mechanism to regulate heteromer formation within the brain. Differential expression levels of CB_1_R do not always correlate with the ability to couple to G-proteins [65], which reinforces the idea that more subtle mechanisms are at play than simple expression. The precise subcellular localization of this heteromeric population of receptors is still not known. However, PLA positive elements could be revealing both pre- and postsynaptic heterodimers, consistent with data showing that CB_1_R and 5-HT_2A_R are colocalized at pre-and postsynaptic levels in different areas of the brain [16,18–27,69,70].

Furthermore, the ICV infusion technique ensured that CB_1_R-5-HT_2A_R heteromers were disrupted by TM interference peptides in key brain areas mediating the observed effects since most of these structures, including the hippocampus, dorsal raphe nucleus, and periaqueductal grey, are in close proximity to the ventricles. Pharmacological targeting of heteromers is of great interest, in part because the GPCR heteromers are unique signaling units with functional properties different from homomers [40,71]. Indeed, our functional data show that costimulation of CB_1_R-5-HT_2A_R heteromers by agonists reduces cell signaling, whereas antagonist binding to one of the receptors blocks the signaling of the interacting receptor (bidirectional cross antagonism). Importantly, we also found that formation of the CB_1_R-5-HT_2A_R heteromers presents a different G-protein coupling, with 5-HT_2A_R coupling to Gi instead of Gq, and a signaling profile different from the single receptors (similar results were previously seen with the mGlu2R-5-HT_2A_R heteromer [72]). In pioneering work, the group of Kobilka has shown that GPCRs are dynamic proteins, adopting a number of conformations through an energy landscape [73]. Ligand or G-protein binding changes the shape of the energy landscape, favoring or disfavoring the intracellular signal. Based on our findings, we propose that in the case of GPCR heteromers one of the protomers allosterically modulates the functional properties of the interacting receptor, and this can be conceptualized using energy landscapes (S7 Fig).

CB_1_R activation by cannabinoids such as THC produces a variety of negative effects, including cognitive impairments [1,2] and anxiogenic- and addictive-like responses [5], which have major consequences in cannabis users and constitute important drawbacks for the use of cannabinoids as therapeutic agents [74]. The genetic, molecular, and pharmacological data presented here demonstrate the requirement for CB_1_R-5-HT_2A_R heteromers for the negative cognitive effects of THC. These heterocomplexes could be potentially modulated in the form of disruption or by their selective pharmacological blockade in order to dissociate the cognitive impairment induced by THC from its beneficial antinociceptive properties.

## Materials and Methods

All the procedures involving animals were performed by observers blind to experimental conditions following standard ethical guidelines (European Communities Directive 86/60-EEC) and were approved by the local ethical committee (Comitè Ètic d'Experimentació Animal-Parc de Recerca Biomèdica de Barcelona, CEEA-PRBB). The PRBB also has Animal Welfare Assurance (#A5388-01, Institutional Animal Care and Use Committee approval date 06/08/2009) granted by the Office of Laboratory Animal Welfare (OLAW) of the United States National Institutes of Health. As of June 2010, the programme of care and use of laboratory animals at the PRBB has the full accreditation from the Association for Assessment and Accreditation of Laboratory Animal Care (AAALAC). Mice were anesthetized with a mixture of ketamine/xylazine and euthanized with carbon dioxide (concentrations between >70% and <100%).

### Animals and Drug Treatment

The 5-HT_2A_R KO and WT littermates were originally generated at Columbia University (US) on a 129S6/SvEv background [29,75]. Animals were backcrossed over at least ten generations onto the inbred C57BL/6J line. Male and female 5-HT_2A_R KO and WT mice were genotyped as previously described [76]. C57BL/6J male, 9-wk-old mice (Charles River L’Arbresle, France) were used for the pharmacological and behavioral experiments. Constitutive CB_1_R KO mice were bred by backcrossing chimeric animals to the C57BL/6J background and crossing heterozygotes [77]. Mice weighing 20–25 g at the beginning of the experiments were initially housed four per cage in a temperature-controlled (21 ± 1°C) and humidity-controlled (55 ± 10%) environment, where food and water were available ad libitum. All the experiments were performed during the light phase of a 12 h light/dark cycle (lights on at 8 a.m. and off at 8 p.m.), except for the WIN 55,212–2 self-administration experiment that was conducted in the dark phase of the cycle. The CB_1_R ligands THC (Pharm GmbH, Frankfurt, Germany) and rimonabant (Sanofi-Aventis Recherche, Montpellier, France) were diluted in 5% ethanol, 5% Cremophor-EL (Sigma-Aldrich), and 90% saline. WIN 55,212–2 (Sigma-Aldrich) was dissolved in one drop of Tween 80 (Sigma-Aldrich) and diluted in physiological saline. For the self-administration experiment, WIN 55,212–2 was administered by intravenous route at 12.5 μg/kg/infusion. The 5-HT_2A_R antagonist, MDL 100,907 (Sigma-Aldrich), was dissolved in saline solution using a drop of Tween 80. Except for WIN 55,212–2, all compounds were administered intraperitoneally (IP) at a volume of 10 ml/kg.

### Behavioral Experiments

In mice, cannabinoids produce the so-called ‘‘tetrad model” of cannabimimetic activity in the same dose range and within the same time frame, consisting of hypolocomotion, hypothermia, antinociception, and catalepsy. Accordingly, we tested the hypolocomotor, hypothermic, and analgesic effects of THC using a complete dose response, as published in previous studies [78]. For memory deficits induced by THC in mice, previous data from our laboratory [79] have revealed significant effects in the object discrimination test only with 3 and 10 mg/kg of THC, which were both tested in the present study. For anxiety-like behavior, it has been shown that THC produces biphasic effects, with lower and higher doses inducing anxiolytic- and anxiogenic-like responses, respectively [3,7]. Accordingly, we measured the anxiolytic-like effect of THC, as well as the consequent increase in social interaction observed, using a low dose (0.3 mg/kg), and the anxiogenic-like response using a high dose (3 mg/kg). The different behavioral effects produced by specific doses of THC suggest that a different level of receptor occupancy is required for each behavioral response [80].

### Determination of Locomotor Activity, Social Interaction, Body Temperature, and Analgesia in Mice

Locomotor responses to acute administration of THC were evaluated by using individual locomotor activity boxes (9 x 20 x 11 cm; Pessac, France) provided with two lines of photocells in a low-luminosity environment (20–25 lux). Mice were placed in the boxes for 45 min (15 min after THC [0.3, 1, 3, and 10 mg/kg] or vehicle administration), and total horizontal activity was analyzed. The social interaction test was performed in an open field (40 x 40 cm) in which mice were habituated for 2 consecutive d in order to maximize the duration of interactions. Next, animals were treated with THC (0.3 mg/kg) or vehicle, and 30 min after the injection, a previously established pair of mice (same strain and size and unfamiliar with each other) was placed into the arena for 10 min. The social interaction test was performed as previously described [81]. Total time spent in active social interactions (defined as sniffing, fighting, chasing, grooming, or crawling under and over each other) was scored from a recorded video. Data were expressed as the amount of interaction time per couple. Body temperature was measured before (basal) and 60 min after THC (1, 3, and 10 mg/kg) or vehicle administration using a lubricated thermo-coupled flexible probe (Panlab, Madrid, Spain) placed into the rectum for 10 s. Data were expressed as change in temperature from basal recording. Antinociceptive effects were evaluated using the tail-immersion and hot-plate tests. The tail-immersion test measures spinal pain responses, which are modulated by descending influences from the brain stem, cerebellum, basal ganglia, and cerebral cortices. All of these structures comprise CB_1_R that play an important role in pain responses [82,83]. The hot-plate test measures supraspinal antinociception, and direct evidence for supraspinal sites of cannabinoid analgesic action has been provided (see Palazzo et al., 2010, for review [83]). Sixty minutes after the injection of THC (1, 3, and 10 mg/kg) or vehicle, the tail-immersion test was carried out, as previously described [84]. The water temperature was maintained at 50 ± 0.5°C using a thermo-regulated water-circulating pump (Clifton, North Somerset, United Kingdom). The latency to a rapid tail flick was registered, and in its absence a 10-s cutoff was used to prevent tissue damage. Subsequently, the hot-plate test was performed, as previously reported [85], 90 min after THC (1, 3, and 10 mg/kg) or vehicle injection. The surface of the plate was kept at 50 ± 0.1°C (Columbus Instruments, Columbus, Ohio, US), and the nociceptive threshold was evaluated by measuring licking and jumping responses. A 5-min cutoff was determined to avoid tissue damage. For both nociceptive models, data were expressed as a percentage of the cutoff latency.

### Determination of the Reinforcing Properties of WIN 55,212–2

Mice were anesthetized with a mixture of ketamine/xylazine (5:1; 0.10 ml/10 g, IP) and implanted with indwelling intravenous Silastic catheters on their right jugular vein as previously described [86]. After surgery, animals were individually housed and allowed to recover for 4 d before initiation of self-administration sessions. The operant model was performed, as previously described [36], in mouse operant chambers (Med Associates, Georgia, Vermont, US) equipped with two nose-pokes, one randomly selected as the active and the other as the inactive nose-poke. Two-hour daily self-administration sessions were conducted consecutively for 12 d. Animals were injected systemically with WIN 55,212–2 in the home cage 24 h before the first self-administration session in order to avoid initial aversive effects. Mice were trained under a fixed ratio 1 (FR1) schedule of reinforcement with a 10-s time-out. Drug self-administration sessions started with a priming injection of the drug, and drug infusion delivery was signaled by the stimulus light together with the pump noise (environmental cues). During the 10-s time-out period, responding on the active hole did not trigger the cue light, and no reward was provided. Each daily session terminated after the delivery of 50 reinforcers or after 2 h, whichever occurred first. Animals needed to achieve the following criteria during three consecutive sessions for the acquisition of self-administration behavior: (1) stable responding on the active hole with <20% deviation from the mean of the total number of reinforcers earned (80% stability); (2) at least 75% responding on the active hole versus the inactive hole (discrimination); and (3) a minimum of eight reinforcers per session. At the end of the self-administration experiment, the patency of the intravenous catheters was evaluated by an infusion of thiopental sodium (0.05 ml at 5 mg/ml) (Braun Medical) through the catheter. Mice that did not show prominent signs of anesthesia within 3 s of the infusion were discarded from the experiment.

### THC-Induced Withdrawal Syndrome Evaluation

Mice were chronically treated with THC (20 mg/kg) or vehicle (twice daily during 5 d intraperitoneally) and received an additional THC (20 mg/kg) or vehicle injection on day 6. Four hours later, the animals were placed in a circular clear plastic observation area (30 cm in diameter and 50 cm in height) for a 15-min period of habituation. Animals were observed for an additional period of 15 min, followed by the administration of rimonabant (10 mg/kg). Somatic signs of withdrawal were evaluated 15 min before and 45 min after rimonabant challenge. The number of wet-dog shakes, front-paw tremors, writhings, and sniffings were counted. Moreover, body tremor, ptosis, teeth chattering, genital licks, hunched posture, and piloerection were scored 1 for appearance or 0 for nonappearance within each 5-min time period. The locomotor activity was rated 0, 1, or 2 (0 for inactivity, 1 for low activity, and 2 for normal activity) over 5-min periods. A global withdrawal score was calculated for each animal by giving each individual sign a relative weight, as previously reported [87].

### Memory Impairments Measurements

The novel object recognition task was performed in a V-maze, as previously described [79]. On the first day, animals were habituated to the maze for 10 min. On the second day, two identical objects in the maze were presented to the animals for 10 min. Immediately after this training period, different doses of THC (3 and 10 mg/kg), which have been reported to produce amnesic-like effects [79], or vehicle were administered to the animals. On the third day, one of the familiar objects was replaced with a novel object, and the time spent exploring both objects was measured. A discrimination index (DI) was calculated as the difference between the times spent exploring either the novel or familiar object divided by the total amount of exploration. DI values above 0.3 were considered to reflect memory retention for the familiar object.

### Anxiety-like Behavior Determination

Anxiety-like behavior was evaluated using the elevated plus maze (EPM), as previously described [88], in a black plastic apparatus with four arms extended from a central platform forming a plus sign. Two opposite arms were delimited by vertical walls (closed arms), whereas the other two opposite arms had unprotected edges (open arms). The maze was elevated 50 cm above the floor and received indirect illumination (70–75 lux in the open arms). A 5-min observation trial was started by placing a mouse on the central platform of the maze with its head facing towards an open arm. The time spent in open and closed arms as well as the number of entrances was recorded. An arm visit was counted when the mouse moved both front paws into the arm. Data are represented as percentage of time spent in the open arms with respect to the total amount of time spent in the open and closed arms. To test the effect of the THC as anxiolytic, the elevated plus maze test was performed 30 min after the administration of 0.3 mg/kg of THC or vehicle, a dose known to induce a decrease in anxiety-like behavior [7]. To test the anxiogenic effect of THC, the elevated plus maze test was performed 5 h after the administration of a dose of 3 mg/kg or vehicle to the animals.

### Electrophysiological Recordings

Immediately after removal from the skull, mouse brains were immersed in an ice-cold artificial cerebrospinal fluid (aCSF composed of NaCl 126 mM, KCl 3.5 mM, NaH_2_PO_4_ 1.2 mM, MgCl_2_ 1.3 mM, CaCl_2_ 2.0 mM, NaHCO_3_ 25 mM, and D-glucose 11 mM) continuously bubbled with carbogen (95% O_2_/5% CO_2_) to maintain pH value at 7.3. Tissue containing the DR nucleus was cut into sections (400 μm thick) in the same ice-cold aCSF using a vibratome. Slices were immediately immersed in oxygenated aCSF and maintained at room temperature (22°C). A single slice was then placed on a nylon mesh in the recording chamber, where it was completely submerged and continuously superfused with oxygenated aCSF (36°C) at a constant flow rate of 2–3 mL/min. Glass microelectrodes filled with 2M NaCl (12–15 MΩ) were used to record the firing activity of DR serotonergic neurons. Neuronal firing was evoked in the otherwise silent neurons by adding the α1-adrenoceptor agonist phenylephrine (3 μM) into the superfusing aCSF [89] and according to previously described criteria [90]; cells were identified as 5-HT neurons. Individual action potentials were amplified by a high-input impedance amplifier (VF 180, BioLogic, Claix, France) and displayed in an oscilloscope connected to an electronic ratemeter, an A/D converter, and a personal computer [91]. The integrated neuronal firing rate was recorded and analyzed in consecutive 10-s samples. Baseline neuronal activity was recorded 5 min before perfusing the brain slices with the different concentrations of THC. Because complete exchange of fluids occurred within 2 min following the arrival of a new solution into the chamber, the duration of each drug application was 3 min. The effects of THC perfusion were evaluated by comparing the mean discharge frequency during the 2 min prior to its application with that recorded at the peak action of the drug. After recovering the firing, neurons were perfused with 5-HT_1A_ receptor agonist ipsapirone (30 nM) to confirm that neurons were in fact 5-HT neurons. Data are expressed as percentage of the baseline firing rate ± SEM.

### Stereotaxic Surgery, Intracerebroventricular (ICV) Infusion of Peptides, and Behavioral Experiments

Animals were anaesthetized with a ketamine/xylazine mixture (5:1; 0.10 ml/10 g, IP) and placed in a stereotaxic apparatus (KOPF Instruments, Tujunga, California). Unilateral cannulae (26 gauge, 8 mm length) were implanted in the right lateral ventricle (AP, -0.2 mm; ML, ±1.0 mm; DV, -2.3 mm from bregma) [92] and then fixed to the skull with dental cement. Mice were housed individually and allowed 3 d of postoperative recovery before experiments began. The ICV injection procedure of interference peptides (TM5, TM6, or TM7) (0.2 μg/2μl) or vehicle was performed at a constant rate of 1 μl/min by using a microinfusion pump (Harvard Apparatus) attached to a 10-μl Hamilton microsyringe (Hamilton, Reno, Nevada) and connected to the ICV cannula through a polyethylene tube (PE-10, Plastics One, Roanoke, Virginia). The tube was removed from the cannula 1 min after the infusion in order to prevent drug reflux. After completion of the experiments, 0.05% methylene blue solution was infused to check the correct position of the cannulae, and data from mice with incorrect placements were discarded. All behavioral tests were performed in the same animals, and 3-d wash-out periods were allowed between tests and ICV infusions. Memory impairments were determined first in the V-Maze. ICV infusions were performed immediately after the training phase and 30 min before THC (3.0 mg/kg IP) or vehicle administration. In order to assure the disruption of the heteromer during the entire time course of THC effects, ICV peptide infusions were repeated 3 h after the first infusion. In concordance with the previous experiment, V-Maze test was performed 24 h after training. Second, anxiety-like responses were carried out in the EPM. ICV infusions were performed 30 min before THC (0.3 mg/kg IP) or vehicle administration, and plus maze observation was conducted 30 min later, in the appropriate conditions to evaluate THC-induced anxiolytic effects. Locomotor activity, body temperature, and analgesia were determined last. Here, ICV infusions were performed 30 min before THC (10 mg/kg IP) or vehicle administration. Fifteen minutes later, animals were placed in locomotor activity boxes for a total of 45 min. Body temperature and tail immersion were performed 60 min after THC or vehicle administration.

### CB_1_R Expression and Endocannabinoid Quantification

Mouse brain samples were dissected, weighted, and immediately frozen at -80°C and kept under these conditions until used. Frozen hippocampal, striatal, cortical, and nucleus accumbens tissues were homogenized using a glass homogenizer in 30 volumes of lysis buffer (50 mM Tris-HCl pH 7.4, 150 mM NaCl, 10% glycerol, 1 mM EDTA, 1 μg/mL aprotinin, 1 μg/mL leupeptine, 1 μg/mL pepstatin, 1 mM phenylmethylsulfonyl fluoride, 1 mM sodium orthovanadate, 100 mM sodium fluoride, 5 mM sodium pyrophosphate, and 40 mM beta-glycerolphosphate) plus 1% Triton X-100. After 10 min incubation at 4°C, samples were centrifuged at 16,000 g for 20 min to remove insoluble debris. Supernatants were collected, and their protein contents were determined by DC-micro plate assay (Bio-Rad, Madrid, Spain), following manufacturer’s instructions. Samples with equal amounts of protein (20 μg per lane) were mixed with denaturing Laemmli loading buffer and separated in a 10% acrylamide gel before electrophoretic transfer onto Immobilon PVDF membrane (Millipore, Darmstadt, Germany). Membranes were blocked for 1 h at room temperature in Tris buffered saline (TBS) with 0.1% Tween-20 (TBS-T) and 5% nonfat milk. Subsequently, membranes were incubated for 2 h with antibodies against CB1R (1:1,000 in TBS-T) (Frontier Science, Ishikari, Japan) and glyceraldehyde-3-phospate dehydrogenase (GAPDH) (1:5,000 in TBS-T with 5% nonfat milk) (Santa Cruz Biotechnology, Santa Cruz, California) as a loading control. Secondary HRP-conjugated antibodies were incubated for 1 h and visualized by enhanced chemiluminescence detection (Luminata Forte, Millipore). The optical density of the relevant immunoreactive band was quantified after acquisition on a Chemi-Doc XRS System (Bio-Rad) by The Quantity One software. Each sample was measured in two independent gels, and the values for CB1R were normalized to the detection of GAPDH in the same samples and expressed as a percentage of the controls. Endocannabinoids were quantified as previously described [93]. Animals were treated with specific inhibitors of the endocannabinoid metabolizing enzymes fatty acid amide hydrolase, URB597 (Biomol-International, Exeter, UK), and monoacylglycerol lipase, JZL184 (Cayman Chemical, Ann Arbor, Michigan). URB597 was injected 1 h before brain extractions, whereas JZL184 was injected 2 h before brain extractions. Both compounds were dissolved in dimethyl sulfoxide (DMSO) (Scharlau Chemie, Barcelona, Spain) and injected IP in a volume of 2 ml/kg. Brain samples were immediately frozen at -80°C and kept under this condition until used. Brain tissue was homogenated with a glass homogenizer in 1 ml 0.02% TFA (pH 3.0) and aliquots of 150 or 20 μl were used for anandamide (AEA) analysis or 2-AG analysis, respectively. Extracts (20 μl) were injected into the liquid chromatography—mass spectrometry (LC-MS-MS) system. An Agilent 6410 triple quadrupole (Agilent Technologies, Wilmington, Delaware) equipped with a 1200 series binary pump, a column oven, and a cooled autosampler (4°C) were used. The chromatographic separation was carried out with a Zorbax 80Å StableBond C8 column (2.1 x 100 mm, 1.8 μm particle size) maintained at 40°C with a mobile phase flow rate of 0.4 ml/min. The composition of the mobile phase was A: 0.1% (v/v) FA in water and B: 0.1% (v/v) FA in acetonitrile. The initial conditions were 40% B. The gradient was increased linearly to 100% B over 4 min, maintained at 100% B for 4 min, and returned to the initial conditions for a further 5.5 min, with a total run time of 13.5 min. The tandem quadrupole mass spectrometer operated on the positive electrospray mode. Desolvation gas temperature of 350°C and a gas flow rate of 10 l/min were used. The pressure of the nebulizer was set at 40 psi and the capillary voltage at 4,000 V. The detection was done by the multiple-reaction monitoring mode, the fragmentor was set at 135 V, and the collision energies were optimized at 12 V for all analytes. The following precursors to product ion transitions were used: m/z 348→62 for AEA, m/z 352→66 for AEA-d4, 379.2→287 for 2- AG, and m/z 384→287 for 2-AG-d5. The quantification was done by isotope dilution based on the deuterated analogues response. The limit of detection on column was 8 pg for AEA and 200 pg for 2-AG.

### Expression Vectors

All human cDNA used were cloned into the pcDNA3.1 vector with geneticin resistance. The cDNA for 5-HT_2A_R was also cloned in a p-CMV hygro destination vector with hygromycin resistance. Sequences encoding amino acid residues 1–155 and 156–238 of YFP Venus protein were subcloned in the pcDNA3.1 vector to obtain the YFP Venus hemi-truncated proteins. The cDNAs for 5-HT_2A_R and dopamine D_1_R were amplified without their stop codons using sense and antisense primers harboring unique EcoRI and Xhol or EcoRI and BamHI sites, respectively. The cDNAs for CB_1_R and adenosine A_1_R were amplified without their stop codons using sense and antisense primers harboring unique EcoRI and KpnI. The amplified fragments were subcloned to be in-frame with restriction sites of pcDNA3.1RLuc (pRLuc-N1 PerkinElmer, Wellesley, Massachusetts), pEYFP-N1 (enhanced yellow variant of GFP, Clontech, Heidelberg, Germany), pcDNA3.1-cVenus, or pcDNA3.1-nVenus vectors to give the plasmids that express proteins fused to RLuc, YFP, or hemi-YFP Venus on the C-terminal end (5-HT_2A_R-RLuc, 5-HT_2A_R-cYFP, D_1_R-RLuc, D_1_R-cYFP, CB_1_R-YFP, CB_1_R-nYFP, A_1_R-nYFP, or A_1_R-YFP). Human β-arrestin II-Rluc6, cloned in the pcDNA3.1 RLuc6 vector (pRLuc-N1 PerkinElmer, Wellesley, Massachusetts) was generously given by Dr. Marian Castro from Santiago de Compostela University, Spain. Expression of constructs was tested by confocal microscopy and the receptor fusion protein functionality by ERK1/2 phosphorylation, as described previously [94–96].

### Cell Culture and Transient Transfection

Human embryonic kidney (HEK-293T) cells obtained from ATCC and HEK-293T cell clones were grown in Dulbecco’s modified Eagle’s medium (DMEM) (Gibco) supplemented with 2 mM L-glutamine, 100 μg/ml sodium pyruvate, 100 U/ml penicillin/streptomycin, MEM Non-Essential Amino Acids Solution (1/100), and 5% (v/v) heat inactivated fetal bovine serum (FBS) (all supplements were from Invitrogen, Paisley, Scotland, UK). As cells expressing CB_1_R, we used a HEK-293T-CB_1_R clone obtained by transfecting HEK-293T cells with CB_1_R cDNA, selected and cultured also in the presence of 200 μg/ml zeocin. To obtain cells expressing 5-HT_2A_R, HEK-293T cells were transiently transfected. HEK-293T cells expressing CB_1_R and 5-HT2_A_R were developed from a HEK-293T-CB_1_R clone by transient transfection of the cDNA corresponding to the 5-HT_2A_R cloned in a p-CMV hygro destination vector and selected in the presence of 200 μg/ml zeocin and 300 μg/ml hygromycin for 3 d. Alternatively, HEK-293T cells were transiently cotransfected with cDNA corresponding to both 5-HT_2A_R and CB_1_R to perform the experiments showed in Fig 4D. Cells growing in 6-well dishes were transiently transfected with the corresponding protein cDNA by the PEI (PolyEthylenImine, Sigma) method. Cells were incubated (4 h) with the corresponding cDNA together with PEI (5.47 mM in nitrogen residues) and 150 mM NaCl in a serum-starved medium. After 4 h, the medium was changed to a fresh complete culture medium. Forty-eight hours after transfection, cells were washed twice in quick succession in HBSS with 10 mM glucose, detached, and resuspended in the same buffer. Cells were maintained at 37°C in an atmosphere of 5% CO_2_. To control the cell number, sample protein concentration was determined using a Bradford assay kit (Bio-Rad, Munich, Germany) using bovine serum albumin dilutions as standards.

### Fluorescence Complementation Assays

HEK-293T were transiently transfected with the cDNA encoding for CB_1_R or A_1_R fused to the YFP Venus N-terminal fragment (n-YFP) and 5-HT_2A_R or D_1_R fused to the YFP Venus C-terminal fragment (c-YFP). After 48 h, cells were treated or not with the indicated TAT-peptides (4 μM) for 4 h at 37°C. To quantify the complemented YFP Venus expression, cells (20 μg protein) were distributed into 96-well microplates (black plates with a transparent bottom, Porvair, King’s Lynn, UK), and fluorescence emission at 530 nm was recorded in a Fluo Star Optima Fluorimeter (BMG Labtechnologies, Offenburg, Germany) equipped with a high-energy xenon flash lamp, using a 10-nm bandwidth excitation filter at 400 nm reading. Protein fluorescence was determined as fluorescence of the sample minus the fluorescence of untransfected cells (basal). Cells expressing 5-HT_2A_R-cVenus and nVenus or CB_1_R-nVenus and cVenus showed similar fluorescence levels to nontransfected cells.

### BRET Assays

HEK-293T cells were transiently cotransfected with a constant amount of expression vectors encoding for proteins fused to RLuc and with increasing amounts of the expression vectors corresponding to proteins fused to YFP (see figure legends). To quantify protein-YFP expression, cells (20 μg protein, around 4,000 cells/well) were distributed in 96-well microplates (black plates with a transparent bottom), and fluorescence was read in a Fluo Star Optima Fluorimeter (BMG Labtechnologies, Offenburg, Germany) equipped with a high-energy xenon flash lamp, using a 10-nm bandwidth excitation filter at 400 nm reading. Fluorescence expression was determined as fluorescence of the sample minus the fluorescence of cells only expressing the BRET donor. For BRET measurements, the equivalent of 20 μg of cell suspension was distributed into 96-well microplates (Corning 3600, white plates; Sigma) and 5 μM coelenterazine H (Molecular Probes, Eugene, OR) was added. The readings were taken 1 min later using a Mithras LB 940. The integration of the signals detected in the short-wavelength filter at 485 nm (440–500 nm), and the long-wavelength filter at 530 nm (510–590 nm) was recorded. To quantify protein-RLuc expression luminescence, readings were also performed 10 min after adding 5 μM coelenterazine H. Fluorescence and luminescence of each sample were measured before every experiment to confirm similar donor expressions (approximately 100,000 bioluminescence units) while monitoring the increase in acceptor expression (1,000 to 40,000 fluorescence units). The net BRET is defined as [(long-wavelength emission) / (short-wavelength emission)]–Cf, where Cf corresponds to [(long-wavelength emission) / (short-wavelength emission)] for the donor construct expressed alone in the same experiment. BRET is expressed as mBU (net BRET x 1,000). Data were fitted to a nonlinear regression equation, assuming a single-phase saturation curve with GraphPad Prism software (San Diego, California, US).

### Immunodetection Assays

For immunocytochemistry, HEK-293T cells stably expressing CB_1_R were grown on glass coverslips and were transiently transfected with the corresponding cDNA. After 48 h of transfection, cells were fixed in 4% paraformaldehyde for 15 min and washed with phosphate-buffered saline (PBS) containing 20 mM glycine to quench the aldehyde groups. After permeabilization with PBS-glycine containing 0.05% Triton X-100 for 5 min, cells were incubated 1 h at room temperature with PBS containing 1% bovine serum albumin and were labeled overnight with the corresponding primary antibody: guinea pig anti-CB_1_R (Frontier Science, Ishikari, Japan) or rabbit anti-CB_1_R antibody (Thermo Scientific, Fremont, California), rabbit anti-5-HT_2A_R antibody (Neuromics, Edina, Minnesota), mouse anti-transferrin antibody (Abcam, Cambridge, UK) or guinea pig anti-D_1_R antibody (Frontier Science, Ishikari, Japan); washed, and stained 2 h with the secondary antibody: chicken anti-rabbit (1:200, Alexa Fluor 594, Invitrogen), goat anti-guinea pig (1:200, Alexa Fluor 488, Invitrogen), or goat anti-mouse (1:200, Alexa Fluor 488, Invitrogen). Samples were rinsed several times and mounted with Mowiol medium (30% Mowiol, Calbiochem, Darmstadt, Germany) and observed using a Leica SP2 confocal microscope (Leica Microsystems, Mannheim, Germany).

### Dynamic Mass Redistribution (DMR) Assays

The global cell signaling profile was measured using an EnSpire Multimode Plate Reader (PerkinElmer, Waltham, Massachusetts, US). This label-free approach uses refractive waveguide grating optical biosensors, integrated into 384-well microplates. Changes in local optical density are measured in a detection zone up to 150 nm above the surface of the sensor. Cellular mass movements induced upon receptor activation are detected by illuminating the underside of the biosensor with polychromatic light and measured as changes in the wavelength of the reflected monochromatic light. These changes are a function of the refraction index. The magnitude of this wavelength shift (in picometers) is directly proportional to the amount of DMR. Briefly, 24 h before the assay, cells were seeded at a density of 10,000 cells per well in 384-well sensor microplates with 30 μl growth medium and cultured for 24 h (37°C, 5% CO2) to obtain 70%–80% confluent monolayers. Previous to the assay, cells were washed twice with assay buffer (HBSS with 20 mM HEPES, pH 7.15) and incubated 2 h in 30 μl per well of assay-buffer with 0.1% DMSO in the reader at 24°C. Hereafter, the sensor plate was scanned, and a baseline optical signature was recorded before adding 10 μl of test compound dissolved in assay buffer containing 0.1% DMSO. Then, DMR responses were monitored for at least 5,000 s. Kinetic results were analyzed using EnSpire Workstation Software v 4.10.

### Brain Slices Preparation

Mice brains were rapidly removed and placed in ice-cold oxygenated (95%O_2_/5%CO_2_) Krebs-HCO_3_- buffer (containing [in mM]: 124 NaCl, 4 KCl, 1.25 KH_2_PO_4_, 1.5 MgCl_2_, 1.5 CaCl_2_, 10 glucose, and 26 NaHCO_3_, pH 7.4). The brains were sliced coronally at 4°C. Slices containing cortex, striatum, nucleus accumbens, or hippocampus (500 μm thick) were kept at 4°C in this Krebs-HCO_3_-buffer during the dissection and were transferred into an incubation tube containing 1 ml of ice-cold Krebs-HCO_3_-buffer. The temperature was raised to 23°C, and after 30 min the medium was replaced by 2 ml of fresh Krebs-HCO_3_-buffer (23°C). The slices were incubated under constant oxygenation (O_2_/CO_2_: 95%/5%) at 30°C for 4–5 h in an Eppendorf Thermomixer (5 Prime, Boulder, Colorado, US). The media was replaced by 200 μl of fresh Krebs-HCO_3_-buffer and incubated for 30 min before the addition of any agent. Slices were treated or not with the indicated ligand for the indicated time. After the indicated incubation period, the solution was discarded, and slices were frozen on dry ice and stored at -80°C.

### Determination of Phospho-ERK 1/2 and Phospho-Akt/PKB in Cells and in Brain Tissue

Transfected HEK-293T cells were cultured in serum-free medium for 16 h before the addition of any compounds. Brain slices were isolated and prepared as indicated above. Cells or slices were either treated or not with the indicated ligands for the times noted, rinsed with ice-cold PBS, and lysed by the addition of 500 μl of ice-cold lysis buffer (50 mM Tris-HCl, pH 7.4, 50 mM NaF, 150 mM NaCl, 45 mM glycerophosphate, 1% Triton X- 100, 20 μM phenyl-arsine oxide, 0.4 mM NaVO_4_, and protease inhibitor mixture). Cellular debris was removed by centrifugation at 13,000 g for 5 min at 4°C, and the amount of protein was quantified by the bicinchoninic acid method using bovine serum albumin dilutions as standard. Equivalent amounts of protein (10 μg) were separated by electrophoresis on a denaturing 10% SDS-polyacrylamide gel and transferred onto PVDF-fluorescence membranes. The membranes were blocked using Odyssey blocking buffer (LI-COR Biosciences), and the membrane was incubated and rocked for 90 min. Membranes were probed with a mouse anti-phospho- ERK1/2 antibody (1:2,500; Sigma, Steinheim, Germany), a rabbit anti-phospho-Ser473-Akt antibody (1/2,500, SAB Signalway Antibody, Pearland, Texas, US), and a rabbit anti-ERK1/2 antibody that recognizes both phosphorylated and nonphosphorylated ERK1/2 (1:40,000; Sigma) simultaneously for 2–3 h. Bands were visualized by the addition of both IRDye 800 (anti-mouse) antibody (1:10,000; Sigma) and IRDye 680 (anti-rabbit) antibody (1:10,000; Sigma) for 1 h. Following incubation, the membranes were washed and scanned by the Odyssey infrared scanner (LI-COR Biosciences). Band densities were measured using the scanner software and then transferred to Excel (Microsoft). Phosphorylated ERK1/2 isoforms or phosphorylated Akt levels were normalized for differences in loading using protein band intensities for total ERK.

### In Situ PLAs in Cells and in Brain Tissue

For proximity ligation assays, mouse brains were fixed by immersion with 4% paraformaldehyde solution for 36 h at 4°C. Samples were then washed in 50 mM Tris-HCl, 0.9% NaCl pH 7.8 buffer (TBS), cryopreserved in a 30% sucrose solution for 48 h at 4°C, and stored at -20°C until sectioning. 30-μm-thick slices were cut sagittally on a freezing cryostat (Leica Jung CM-3000) and mounted on slide glass. Brain slices were thawed at 4°C, washed in TBS, permeabilized with TBS containing 0.01% Triton X-100 for 10 min, and successively washed with TBS. Cells stably expressing CB_1_R and transfected with the corresponding cDNA were grown on glass coverslips and were fixed in 4% paraformaldehyde for 15 min, washed with PBS containing 20 mM glycine, permeabilized with the same buffer containing 0.05% Triton X-100, and successively washed with PBS. Heteromers were detected using the Duolink II in situ PLA detection Kit (OLink; Bioscience, Uppsala, Sweden) and following the instructions of the supplier. To detect CB_1_R-5-HT_2A_R heteromers, a mixture of equal amounts of rabbit anti-CB_1_R antibody (Thermo Scientific, Fremont, California) directly linked to a plus PLA probe and rabbit anti-5-HT_2A_R antibody (Neuromics, Edina, MN) directly linked to a minus PLA probe was used. PLA probe was linked to the antibodies following the instructions of the supplier. To detect CB_1_R-transferrin receptor or CB_1_R-D_1_R heteromers, a mixture of equal amounts of rabbit anti-CB_1_R antibody and mouse anti-transferrin antibody (Abcam, Cambridge, UK) or guinea pig anti-D_1_R antibody (Frontier Institute, Ishikari, Hokkaido, Japan) were used and incubated with anti-rabbit plus and anti-mouse minus PLA probes or anti-rabbit plus and anti-guinea pig minus PLA probes, respectively. Cells and slices were mounted using the mounting medium with DAPI. The samples were observed in a Leica SP2 confocal microscope (Leica Microsystems, Mannheim, Germany) equipped with an apochromatic 63X oil-immersion objective (N.A. 1.4), and a 405 nm and a 561 nm laser line. For each field of view, a stack of two channels (one per staining) and 9 to 15 Z stacks with a step size of 1 μm were acquired. Images were opened and processed with Image J confocal. After image processing, the red channel was depicted in green color to facilitate detection on the blue-stained nucleus and to maintain the color intensity constant for all images. In tissue, a quantification of cells containing one or more green spots versus total cells (blue nucleus) was determined considering a total of 1,500–3,000 cells from 4–12 different fields within each region from three different animals. In cells, the ratio r (number of red spots/number of cells containing spots) was determined considering a total of 1,500–3,000 cells from 8–12 different fields. In both cases, the ImageJ confocal program using the Fiji package (http://pacific.mpi-cbg.de/) was used. Nuclei and green spots were counted on the maximum projections of each image stack. After getting the projection, each channel was processed individually. The nuclei were segmented by filtering with a median filter, subtracting the background, enhancing the contrast with the contrast limited adaptive histogram equalization (CLAHE) plug-in, and finally applying a threshold to obtain the binary image and the regions of interest (ROIs) around each nucleus. Green spot images were also filtered and thresholded to obtain the binary images. Green spots were counted in each of the ROIs obtained in the nuclei images.

### cAMP Production and Arrestin Recruitment

For cAMP production, homogeneous time-resolved fluorescence energy transfer (HTRF) assays were performed using the Lance Ultra cAMP kit (PerkinElmer, Waltham, Massachusetts, US), based on competitive displacement of a europium chelate-labelled cAMP tracer bound to a specific antibody conjugated to acceptor beads. We first established the optimal cell density for an appropriate fluorescent signal. This was done by measuring the TR-FRET signal determined as a function of forskolin concentration using different cell densities. The forskolin dose-response curves were related to the cAMP standard curve in order to establish which cell density provides a response that covers most of the dynamic range of cAMP standard curve. Cells (1,000 cells/well) growing in medium containing 50 μM zardeverine were pretreated with the antagonists or the corresponding vehicle in white ProxiPlate 384-well microplates (PerkinElmer) at 25°C for 20 min and stimulated with agonists for 15 min before adding 0.5 μM forskolin or vehicle and incubating for an additional 15-min period. Fluorescence at 665 nm was analyzed on a PHERAstar Flagship microplate reader equipped with an HTRF optical module (BMG Lab technologies, Offenburg, Germany). Arrestin recruitment was determined using BRET experiments as described above in HEK-293T expressing β-arrestin II-Rluc, 5-HT_2A_R-YFP and CB_1_R after the indicated treatment with ligands.

### Calcium Signalling

To determine calcium release, cells stably expressing CB_1_R were transfected with the cDNA for 5-HT_2A_R and 4 μg of GCaMP6 calcium sensor [97] using lipofectamine. 48 h after transfection, cells were incubated (0.2 mg of protein/ml in 96-well black, clear bottom microtiter plates) with Mg^+2^-free Locke’s buffer pH 7.4 (154 mM NaCl, 5.6 mM KCl, 3.6 mM NaHCO_3_, 2.3 mM CaCl_2_, 5.6 mM glucose, and 5 mM HEPES) supplemented with 10 μM glycine. Then, receptor ligands were added as indicated. Fluorescence emission intensity of GCaMP6 was recorded at 515 nm upon excitation at 488 nm on an EnSpire Multimode Plate Reader (PerkinElmer, Boston, Massachusetts, US) for 335 s every 15 s and 100 flashes per well.

### Statistical Analyses

The behavioral data are presented as mean + SEM and were analyzed using one-, two-, or three-way ANOVA when appropriate with genotype (WT and 5-HT_2A_R KO mice) and treatment (vehicle and THC) or pretreatment (vehicle and MDL 100,907) and treatment (vehicle and THC) as between-subjects factors, followed by post hoc comparisons when appropriate. The WIN 55,212–2 self-administration data were analyzed using three-way ANOVA with genotype (WT and 5-HT_2A_R KO mice) as between-subjects factor and nose-poke (active and inactive) and day of session as within-subjects factors. Statistical significance was set at *p* < 0.05 level. All tests were two-sided. The in vitro data are represented as mean + SEM and were analyzed using unpaired Student’s *t* test or one-way ANOVA followed by Bonferroni post-hoc tests when appropriate.

## Supporting Information

S1 Data Excel spreadsheet containing, in separate sheets, the underlying numerical data for figure panels Fig 1A–1C, Fig 1E–1I, Fig 2A–2G, Fig 3A–3D, Fig 4A–4B, Fig 4D, Fig 5A–5D, Fig 5F–5H, Fig 7B–7D, Fig 8B, Fig 9A–9D, Fig 10A–10D, Fig 10F–10I, S1B–S1E Fig, S2A–S2D Fig, S3A–S3F Fig, S5A–S5H Fig, S6A–S6D Fig, S9 Fig, and S11A–S11J Fig.
(XLSX)

S1 Fig CB_1_R protein levels and endocannabinoid quantification in WT and 5-HT_2A_R KO mice. In (A) western blots are represented showing the presence of CB_1_R in the cortex, striatum, nucleus accumbens, and hippocampus of CB_1_R WT, but not of KO mice. In (B and C), the percentage of CB_1_R protein with respect to GAPDH was reduced in the hippocampus (B) and cerebellum (C) of WT and 5-HT_2A_R KO mice repeatedly treated with THC, and this effect was significantly greater in the hippocampus of KO animals, but not in the cerebellum (*n* = 5–6). Representative western blot bands are depicted in the lower panels. *** *p* < 0.001 versus vehicle; # *p* < 0.05, ## *p* < 0.01 versus WT animals. In (D and E), the levels of anandamide (D) were significantly reduced in 5-HT_2A_R KO mice as compared to WT mice, while 2-arachidonoylglycerol (2-AG) levels (E) were similar in both genotypes (*n* = 7–8). * *p* < 0.05 versus WT animals. The statistical analyses used and their corresponding F and *p*-values are shown in S2 Table.
(TIFF)

S2 Fig Selectivity of CB_1_R and 5-HT_2A_R agonists and antagonists. DMR analysis was performed in HEK-293Tcells expressing CB_1_R (A and B) or 5-HT_2A_R (C and D). In (A and C), cells were stimulated with increasing concentrations of CB_1_R agonists WIN 55,212–2 (WIN) or THC (A) or 5-HT_2A_R agonists DOI or serotonin (C). In (B and D), cells were pretreated for 20 min with medium, the CB_1_R antagonist rimonabant (1 μM, RIM), or the 5-HT_2A_R antagonist MDL 100,907 (300 nM, MDL) before stimulation with WIN 55,212–2 (WIN), or DOI. In all cases, the resulting picometer shifts of reflected light wavelength (pm) were monitored over time. Each curve is the mean of a representative optical trace experiment carried out in triplicates.
(TIF)

S3 Fig CB_1_R are associated to a Gi protein and 5-HT_2A_R are associated to a Gq protein when expressed alone. HEK-293T cells expressing CB_1_R (A and B) or 5-HT_2A_R (C–F) were used. Cells were not treated (control) or treated overnight with 10 ng/ml pertussis toxin (PTX, A–D), treated 1 h (B and D) or overnight (A and C) with 100 ng/ml cholera toxin (CTX), or treated for 30 min with 1 μM of the Gq protein inhibitor YM-254890 (B–E). In (A, C, and E), the dynamic mass redistribution analysis was performed in (A) control cells (red line), cells treated with PTX (green line) or CTX (blue line) stimulated with 50 nM WIN 55,212–2 or (C and E) control cells (black lines), cells treated with PTX (purple line C) or CTX (yellow line C), or cells treated with YM-254890 (orange line E), stimulated with 100 nM DOI. The resulting picometer shifts of reflected light wavelength (pm) were monitored over time. Each curve is the mean of a representative optical trace experiment carried out in triplicates. In (B and D), cAMP production was determined after stimulation with 100 nM DOI or 100 nM WIN 55,212–2 (WIN) in the absence or in the presence of 0.5 μM forskolin. Values (cAMP produced in each condition minus basal stimulation in the absence of forskolin or agonists) represent mean ± SEM of *n* = 3–4 and are expressed as the percentage of the forskolin-treated cells in control conditions (120–150 pmols cAMP/106 cells). For cells treated with forskolin, one-way ANOVA followed by a Dunnett’s multiple comparison post hoc test showed a significant effect over the forskolin-alone effect in each condition (*** *p* < 0.001). Basal cAMP concentration was very similar in all conditions. In (F), intracellular calcium release was monitored in untreated HEK-293T cells expressing 5-HT2AR (black curve) or pretreated with the 5-HT2AR antagonist MDL 100,907 (300 nM, orange curve) 30 min before stimulation with 100 nM DOI. Values are mean ± SEM of *n* = 3.
(TIF)

S4 Fig Negative controls for proximity ligation assays in transfected cells. PLAs were performed in HEK-293T cells stably expressing CB_1_R (A) or stably expressing CB_1_R and transfected with 2 μg cDNA corresponding to dopamine D_1_ receptors (B). PLA was performed using anti-CB_1_R antibody and anti-transferrin receptor antibodies (A) or anti-CB_1_R antibody and anti-D_1_R antibodies (B) as primary antibodies. Confocal immunocytochemistry images are shown at top in (A and B) showing colocalization (yellow) between CB_1_R (red) and transferrin (green) or D_1_ (green) receptors. In (C), PLA was performed in a 1:1 mixture of cells only expressing CB_1_R or 5-HT_2A_R using anti-CB_1_R and anti-5-HT_2A_R antibodies. Confocal microscopy images (superimposed sections) are shown in which green spots corresponding to the heteromers are absent in all cases. Cell nuclei were stained with DAPI (blue). Scale bars = 20 μm
(TIF)

S5 Fig Time and dose response for agonist-induced signaling in cells expressing CB_1_R or 5-HT_2A_R alone. HEK-293Tcells expressing CB_1_R (A, B, E, and F) or 5-HT_2A_R (C, D, G, and H) were stimulated at increasing times (min) with 100 nM WIN 55,212–2 (WIN) (A and E) or for 5 min with increasing WIN 55,212–2 concentrations (μM) (B and F) or were stimulated for increasing times (min) with 100 nM DOI (C and G) or for 5 min with increasing DOI concentrations (μM) (D and H), and quantification of phosphorylated ERK 1/2 (A, B, C, and D) or Akt (E, F, G, and H) was determined by western blot. Values, expressed as percentage of basal (nonagonist treated cells), were mean ± SEM of *n* = 3–6.
(TIF)

S6 Fig Time response of agonist-induced signaling in cells coexpressing CB_1_R and 5-HT_2A_R and determination of ligand specificity in cells expressing single receptors. In (A and B), HEK-293T cells expressing 5-HT_2A_R and CB_1_R were stimulated at increasing times (min) with 100 nM WIN 55,212–2 (WIN), 100 nM DOI, or both. In (C and D), HEK-293Tcells expressing CB_1_R (C) or 5-HT_2A_R (D) were preincubated or not with rimonabant (1 μM, RIM) or MDL 100,907 (300 nM, MDL) for 15 min and then stimulated for 5 min with WIN 55,212–2 (100 nM, WIN) or DOI (100 nM). Quantification of phosphorylated ERK 1/2 or Akt was determined by western blot. Values, expressed as percentage of basal (nonagonist or antagonist treated cells), were mean ± SEM of *n* = 3–6. One-way ANOVA followed by Bonferroni post hoc tests showed a significant (* *p* < 0.05, *** *p* < 0.001) effect over basal or no significant effect (*p* > 0.04) of the antagonist plus agonist treatment over the agonist treatment.
(TIF)

S7 Fig Dynamic properties of the CB_1_R-5-HT_2A_R heteromer. (A) Intracellular view of the CB_1_R-5-HT_2A_R heteromer (blue and green protomers) bound to DOI (green surface) and rimonabant (red surface), modeled from the crystal structure of the μ-opioid receptor (PDB id 4DKL) [1]. TMs 5 and 6 of rhodopsin (light brown, 1GZM) [2], the β_2_-adrenergic receptor (yellow, 2RH1) [3], and the β_2_-adrenergic receptor in complex with Gs (orange, 3SN6) [4] are superimposed on the 5-HT_2A_R. This superimposition shows that the conformational equilibrium of GPCRs primarily consists of different conformations of TMs 5 and 6, opening or closing an intracellular cavity for binding of the G-protein with minimal movement of the other TMs. Agonists stabilize conformations of TMs 5 and 6 that facilitate the opening of this intracellular cavity (TMs 5 and 6 in orange), whereas inverse agonists (antagonists) stabilize other conformations of these helices that close this cavity (TMs 5 and 6 in green, light brown, or yellow). TMs 5 and 6 of protomer A, in the closed conformations, can interact with TMs 5 and 6 of protomer B (via a four-helix bundle, green and blue TMs 5 and 6) as observed in the crystal of the μ-opioid receptor. In this assembly, both protomers are locked in the closed conformation since the opening of TMs 5 and 6 for G-protein binding is not feasible. (B) Many GPCRs can bind their G-protein in the absence of an agonist, showing basal activity [5]. This suggests that GPCRs are dynamic proteins that permit rapid small-scale structural fluctuations and pass through an energy landscape to adopt a number of conformations, ranging from inactive to active [6]. The transition probability from one state to another depends on the energy difference between both states and the energy barrier between them. Ligand binding to a monomer (left panel, adapted from [7]) changes the shape of the energy landscape relative to the unliganded landscape (black line), in such a manner that inverse agonists/antagonists (in red) stabilize inactive conformations (red line), agonists (in green) stabilize intermediate conformations, and the final formation of the agonist-receptor-G-protein complex stabilizes active conformations (green line). Based on our findings, we propose that in the case of receptor heteromers (right panel) one of the protomers allosterically modulates the energy landscape of the interacting receptor. Relative to the unliganded landscape (black line), in which both TM 5 and TM 6 of the receptor (see above) and the TM contacts between protomers are in a dynamic equilibrium of conformations, antagonist binding stabilizes the inactive conformations of the receptor (closed cavity) plus the four-helix TM 5 and TM 6 bundle (red line). Importantly, this energy minimum of the antagonist-bound receptor is more stable in heteromers than in monomers because of the additional formation of the four-helix bundle. These low free-energy states of the antagonist-bound heteromer (inactive states) impede the possibility that agonist binding to the other protomer would reach the energy minima of the active states (blue line), leading to cross antagonism. Agonist binding to one protomer stabilizes the active conformations of TM 5 and TM 6 (open cavity), TM contacts between protomers that facilitate these active conformations, and the binding of the G-protein to the active protomer (dark green line). Simultaneous activation of both protomers by their corresponding agonists leads to energy minima less stable than activation of a single protomer (light green line), most probably because of a steric clash of the active conformations of TM 5 and TM 6 in both protomers, which results in a reduction of cell signaling.
(TIF)

S8 Fig Effects of disrupting peptides on CB_1_R and 5-HT_2A_R expression and colocalization. Immunocytochemistry experiments were performed in cells expressing CB_1_R and 5-HT_2A_R preincubated for 4 h with vehicle (top panels) or with 4 μM of CB_1_R TM 5, TM 6, or TM 7 interference peptides using guinea pig anti-CB_1_R (Frontier Science, Ishikari, Japan) and rabbit anti-5-HT_2A_R antibody (Neuromics, Edina, Minnesota). Confocal microscopy images showing colocalization (yellow) between CB_1_R (green) and 5-HT_2A_R (red) are shown. Scale bars = 20 μm.
(TIF)

S9 Fig Effects of disrupting peptides on CB_1_R-5-HT_2A_R heteromerization. HEK-293T cells transfected with 4 μg of cDNA corresponding to both 5-HT_2A_R-cYFP and CB_1_R-nYFP were treated for 4 h with vehicle or 4 μM of CB_1_R TM 5, TM 6, or TM 7 interference peptides prior to the fluorescence determination at 530 nm. One-way ANOVA followed by Bonferroni post hoc tests showed a significant (* *p* < 0.05, *** *p* < 0.001) effect over basal fluorescence (1,500–2,000 fluorescence units in nontransfected cells) or compared to peptide treatment over the vehicle treatment (^##^ *p* < 0.01, ^###^ *p* < 0.001).
(TIF)

S10 Fig Negative controls for proximity ligation assays in brain slices. PLAs were performed using slices of mouse cortex (somatomotor layers 1, 2, and 3), caudate-putamen (striatum), hippocampus CA3, or nucleus accumbens (NaC) from WT (A) and 5-HT_2A_R KO (B) mice, using anti-CB_1_R and anti-dopamine D_1_ receptor antibodies as primary antibodies. Confocal microscopy images (superimposed sections) are shown in which green spots corresponding to the heteromers are absent in all panels. In all cases, cell nuclei were stained with DAPI (blue). Scale bars = 20 μm.
(TIFF)

S11 Fig Effects of TM 6 and TM 7 interference peptides on THC-induced behavioral responses in WT and 5-HT_2A_R OK mice. Pretreatment with TM 6, but not with TM 7, peptides (0.2 μg/ 2 μl ICV) blocked the memory deficits and anxiolytic-like behavior induced by THC (3 and 0.3 mg/kg, respectively) in WT mice (A and B), but neither TM 6 nor TM 7 peptides modified these effects in 5-HT_2A_R KO mice (F and G) (*n* = 4–6). Hypolocomotion, hypothermia, and analgesia induced by THC (10 mg/kg) were not altered by pretreatment with TM 6 or TM 7 peptides in WT mice (C–E) or in 5-HT_2A_R KO animals (H–J) (*n* = 7–11). All data represent mean + SEM. * *p* < 0.05, ** *p* < 0.01, *** *p* < 0.001 versus vehicle. The statistical analyses used and their corresponding F and p values are shown in S2 Table.
(TIF)

S1 Table Statistical analyses used for the behavioral data presented in Figs 1, 2, and 10. Corresponding F and *p*-values are shown.
(DOCX)

S2 Table Statistical analyses used for the behavioral data presented in S1 Fig and S9 Fig. Corresponding F and *p*-values are shown.
(DOCX)

## Abbreviations

2-AG: 2-arachidonoylglycerol
5-HT: serotonin
5-HT_2A_R: serotonin 2A receptors
A_1_: adenosine receptor type 1
aCSF: artificial cerebrospinal fluid
AEA: anandamide
Akt: protein kinase B
BiFC: bimolecular fluorescence complementation
BRET: bioluminescent resonance energy transfer
cAMP: cyclic adenosine monophosphate
CB_1_R: cannabinoid receptor type 1
CLAHE: contrast limited adaptive histogram equalization
CTX: cholera toxin
D_1_: dopamine receptor type 1
DAPI: 4',6-diamidino-2-phenylindole
DI: discrimination index
DMEM: Dulbecco’s modified Eagle’s medium
DMR: dynamic mass redistribution
DMSO: dimethyl sulfoxide
DOI: (±)-1-(2,5-dimethoixy-4-odophenyl)-2-aminopropane
DR: dorsal raphe
EPM: elevated plus maze
ERK: extracellular signal-regulated kinase
FBS: fetal bovine serum; FR1, fixed ratio 1
GABA: γ-aminobutyric acid
GPCR: G protein-coupled receptor
GWS: global withdrawal score
HEK: human embryonic kidney
HIV: human immunodeficiency virus
HTRF: homogeneous time-resolved fluorescence
ICV: intracerebroventricular
IP: intraperitoneally
KO: knockout
LC-MS-MS: liquid chromatography—mass spectrometry
mBU: milli BRET unit
mGlu2R: metabotropic glutamate receptor type 2
PBS: phosphate-buffered saline
PLA: proximity ligation assay
PTX: pertussis toxin
RIM: rimonabant
Rluc: renilla luciferase
ROI: region of interest
SD: standard deviation
SEM: standard error of the mean
TBS: Tris-buffered saline
THC: delta9-tetrahydrocannabinol
TM: transmembrane helix
TR-FRET: time-resolved fluorescent resonance energy transfer
VEH: vehicle
WT: wild type
YFP: yellow fluorescent protein
