# Supplementary figures and images for: Cognitive Impairment Induced by Delta9-tetrahydrocannabinol Occurs through Heteromers between Cannabinoid CB1 and Serotonin 5-HT2A Receptors
Source: PLoS Biol. 2015 Jul 9;13(7):e1002194. doi: 10.1371/journal.pbio.1002194 (PMC4497644; doi:10.1371/journal.pbio.1002194)

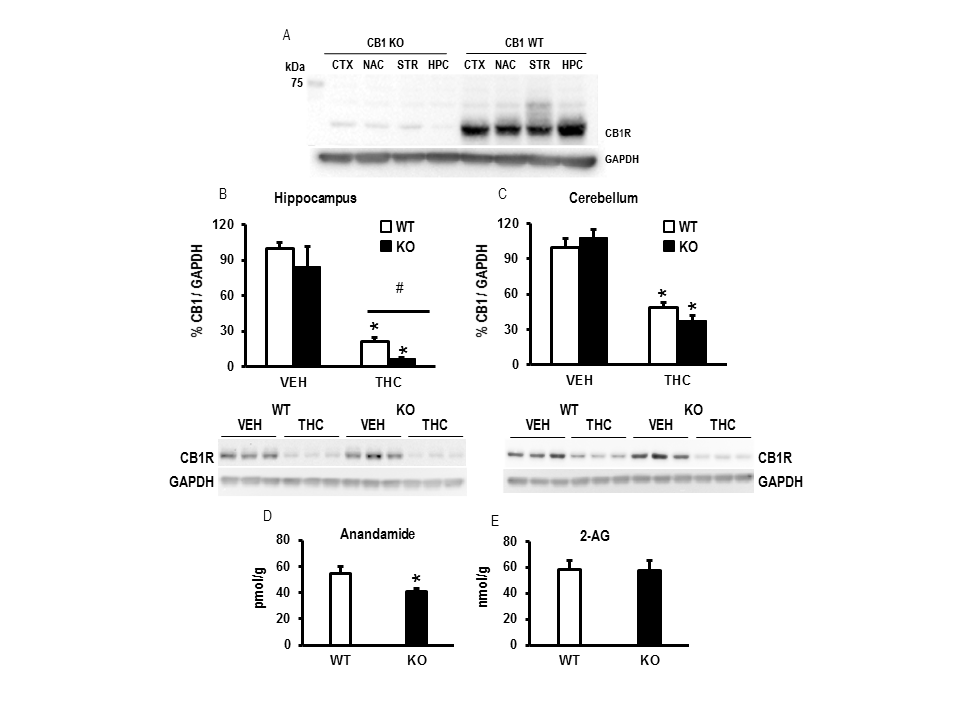

Supplement: S1 Fig — In (A) western blots are represented showing the presence of CB1R in the cortex, striatum, nucleus accumbens, and hippocampus of CB1R WT, but not of KO mice. In (B and C), the percentage of CB1R protein with respect to GAPDH was reduced in the hippocampus (B) and cerebellum (C) of WT and 5-HT2AR KO mice repeatedly treated with THC, and this effect was significantly greater in the hippocampus of KO animals, but not in the cerebellum (n = 5–6). Representative western blot bands are depicted in the lower panels. *** p < 0.001 versus vehicle; # p < 0.05, ## p < 0.01 versus WT animals. In (D and E), the levels of anandamide (D) were significantly reduced in 5-HT2AR KO mice as compared to WT mice, while 2-arachidonoylglycerol (2-AG) levels (E) were similar in both genotypes (n = 7–8). * p < 0.05 versus WT animals. The statistical analyses used and their corresponding F and p-values are shown in S2 Table. (TIFF) [file pbio.1002194.s002.tiff]

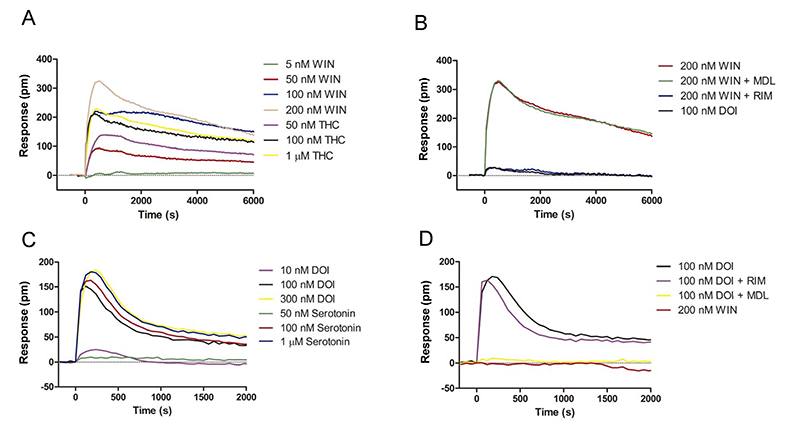

Supplement: S2 Fig — DMR analysis was performed in HEK-293Tcells expressing CB1R (A and B) or 5-HT2AR (C and D). In (A and C), cells were stimulated with increasing concentrations of CB1R agonists WIN 55,212–2 (WIN) or THC (A) or 5-HT2AR agonists DOI or serotonin (C). In (B and D), cells were pretreated for 20 min with medium, the CB1R antagonist rimonabant (1 μM, RIM), or the 5-HT2AR antagonist MDL 100,907 (300 nM, MDL) before stimulation with WIN 55,212–2 (WIN), or DOI. In all cases, the resulting picometer shifts of reflected light wavelength (pm) were monitored over time. Each curve is the mean of a representative optical trace experiment carried out in triplicates. (TIF) [file pbio.1002194.s003.tif]

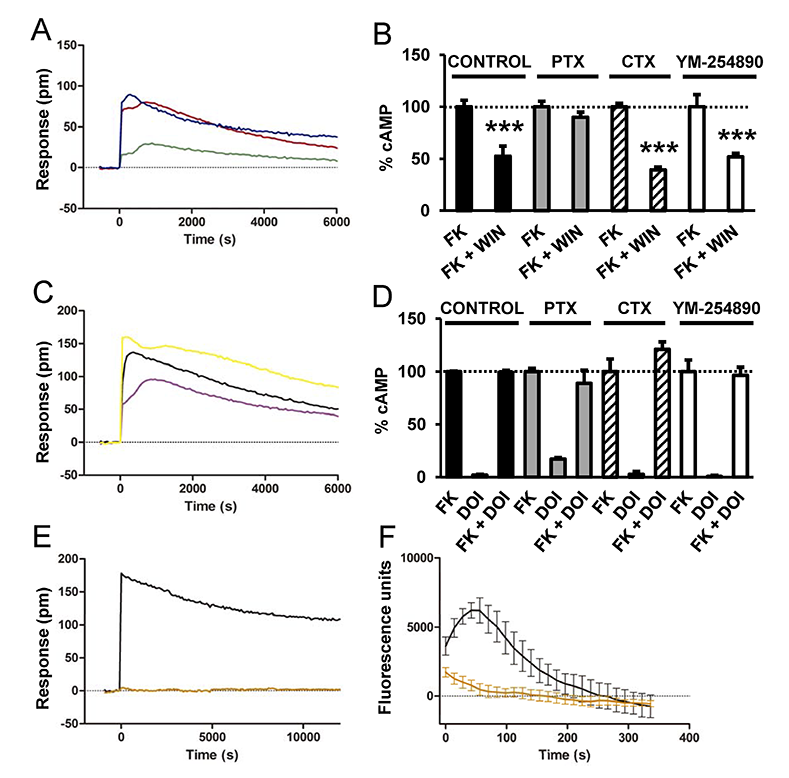

Supplement: S3 Fig — HEK-293T cells expressing CB1R (A and B) or 5-HT2AR (C–F) were used. Cells were not treated (control) or treated overnight with 10 ng/ml pertussis toxin (PTX, A–D), treated 1 h (B and D) or overnight (A and C) with 100 ng/ml cholera toxin (CTX), or treated for 30 min with 1 μM of the Gq protein inhibitor YM-254890 (B–E). In (A, C, and E), the dynamic mass redistribution analysis was performed in (A) control cells (red line), cells treated with PTX (green line) or CTX (blue line) stimulated with 50 nM WIN 55,212–2 or (C and E) control cells (black lines), cells treated with PTX (purple line C) or CTX (yellow line C), or cells treated with YM-254890 (orange line E), stimulated with 100 nM DOI. The resulting picometer shifts of reflected light wavelength (pm) were monitored over time. Each curve is the mean of a representative optical trace experiment carried out in triplicates. In (B and D), cAMP production was determined after stimulation with 100 nM DOI or 100 nM WIN 55,212–2 (WIN) in the absence or in the presence of 0.5 μM forskolin. Values (cAMP produced in each condition minus basal stimulation in the absence of forskolin or agonists) represent mean ± SEM of n = 3–4 and are expressed as the percentage of the forskolin-treated cells in control conditions (120–150 pmols cAMP/106 cells). For cells treated with forskolin, one-way ANOVA followed by a Dunnett’s multiple comparison post hoc test showed a significant effect over the forskolin-alone effect in each condition (*** p < 0.001). Basal cAMP concentration was very similar in all conditions. In (F), intracellular calcium release was monitored in untreated HEK-293T cells expressing 5-HT2AR (black curve) or pretreated with the 5-HT2AR antagonist MDL 100,907 (300 nM, orange curve) 30 min before stimulation with 100 nM DOI. Values are mean ± SEM of n = 3. (TIF) [file pbio.1002194.s004.tif]

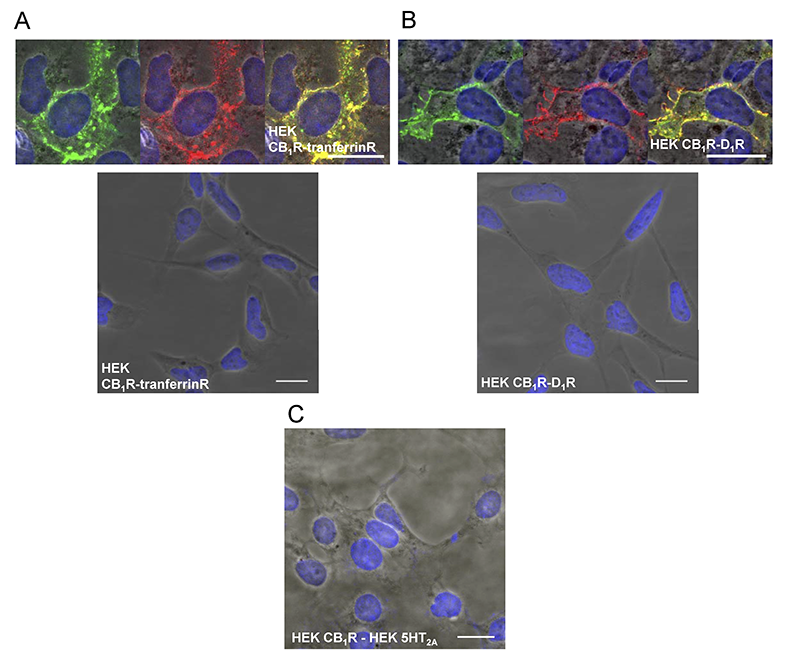

Supplement: S4 Fig — PLAs were performed in HEK-293T cells stably expressing CB1R (A) or stably expressing CB1R and transfected with 2 μg cDNA corresponding to dopamine D1 receptors (B). PLA was performed using anti-CB1R antibody and anti-transferrin receptor antibodies (A) or anti-CB1R antibody and anti-D1R antibodies (B) as primary antibodies. Confocal immunocytochemistry images are shown at top in (A and B) showing colocalization (yellow) between CB1R (red) and transferrin (green) or D1 (green) receptors. In (C), PLA was performed in a 1:1 mixture of cells only expressing CB1R or 5-HT2AR using anti-CB1R and anti-5-HT2AR antibodies. Confocal microscopy images (superimposed sections) are shown in which green spots corresponding to the heteromers are absent in all cases. Cell nuclei were stained with DAPI (blue). Scale bars = 20 μm (TIF) [file pbio.1002194.s005.tif]

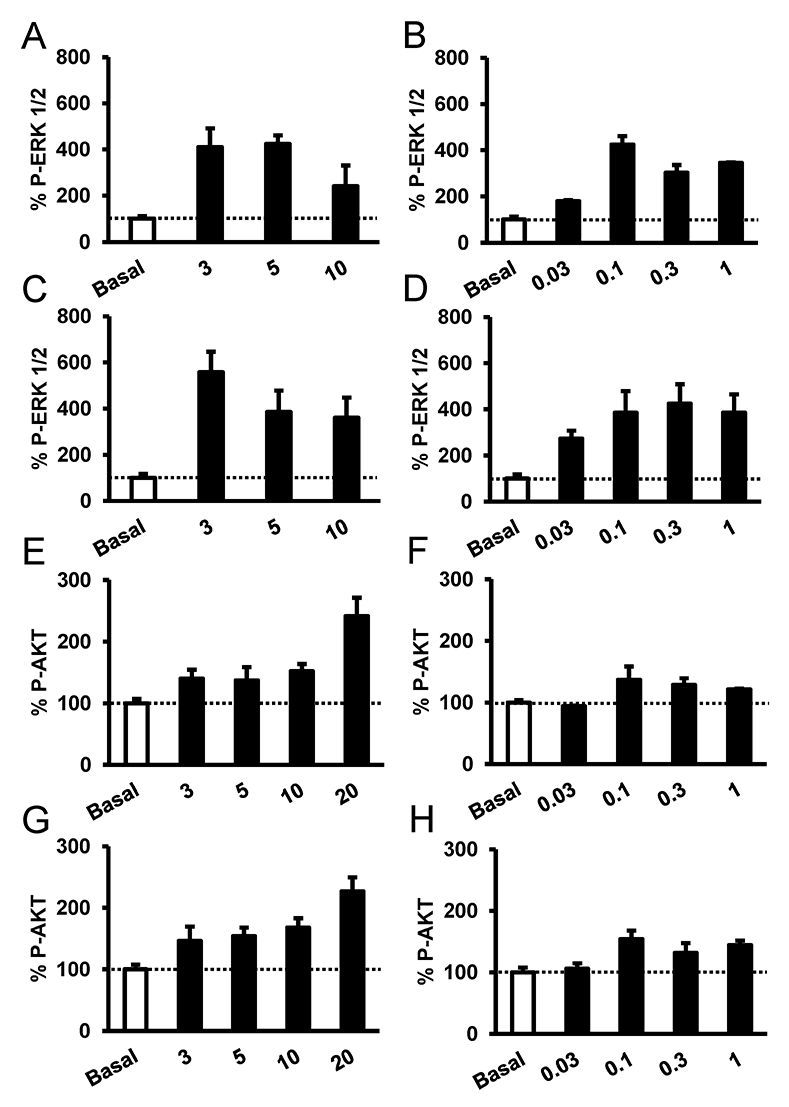

Supplement: S5 Fig — HEK-293Tcells expressing CB1R (A, B, E, and F) or 5-HT2AR (C, D, G, and H) were stimulated at increasing times (min) with 100 nM WIN 55,212–2 (WIN) (A and E) or for 5 min with increasing WIN 55,212–2 concentrations (μM) (B and F) or were stimulated for increasing times (min) with 100 nM DOI (C and G) or for 5 min with increasing DOI concentrations (μM) (D and H), and quantification of phosphorylated ERK 1/2 (A, B, C, and D) or Akt (E, F, G, and H) was determined by western blot. Values, expressed as percentage of basal (nonagonist treated cells), were mean ± SEM of n = 3–6. (TIF) [file pbio.1002194.s006.tif]

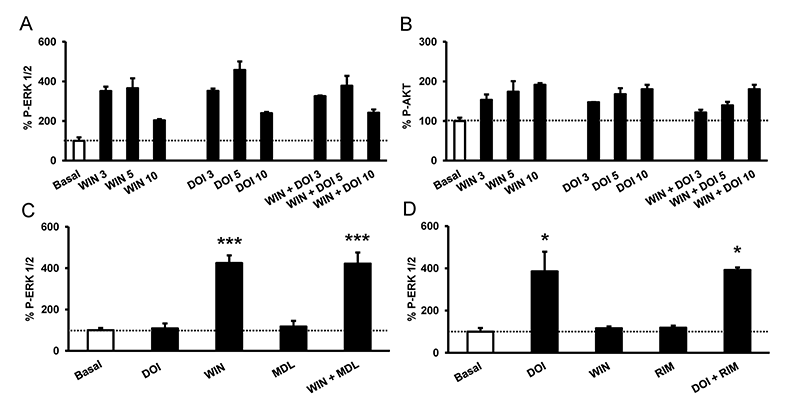

Supplement: S6 Fig — In (A and B), HEK-293T cells expressing 5-HT2AR and CB1R were stimulated at increasing times (min) with 100 nM WIN 55,212–2 (WIN), 100 nM DOI, or both. In (C and D), HEK-293Tcells expressing CB1R (C) or 5-HT2AR (D) were preincubated or not with rimonabant (1 μM, RIM) or MDL 100,907 (300 nM, MDL) for 15 min and then stimulated for 5 min with WIN 55,212–2 (100 nM, WIN) or DOI (100 nM). Quantification of phosphorylated ERK 1/2 or Akt was determined by western blot. Values, expressed as percentage of basal (nonagonist or antagonist treated cells), were mean ± SEM of n = 3–6. One-way ANOVA followed by Bonferroni post hoc tests showed a significant (* p < 0.05, *** p < 0.001) effect over basal or no significant effect (p > 0.04) of the antagonist plus agonist treatment over the agonist treatment. (TIF) [file pbio.1002194.s007.tif]

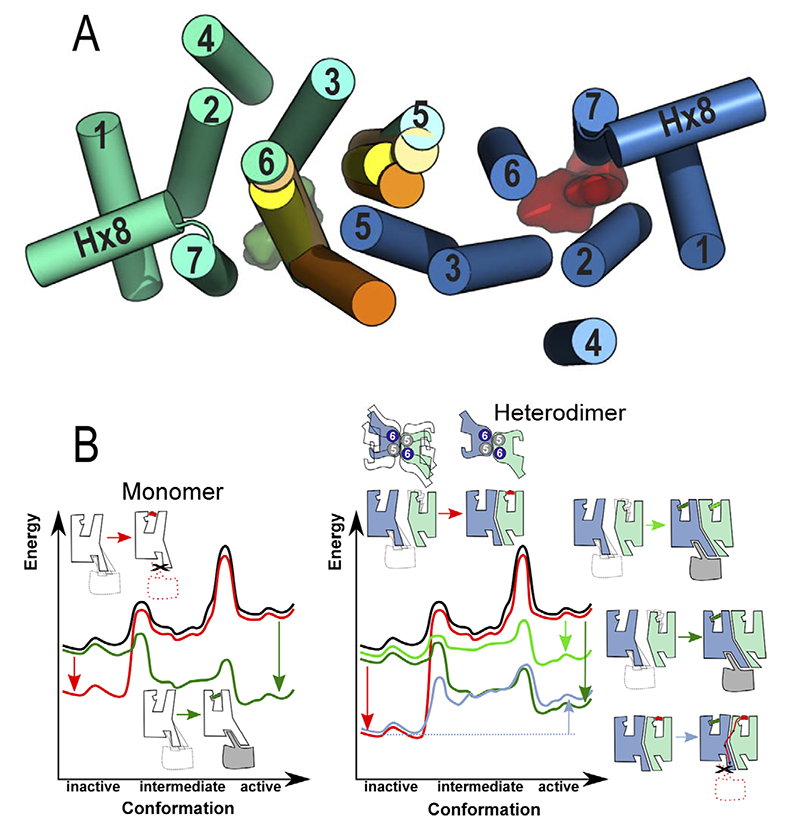

Supplement: S7 Fig — (A) Intracellular view of the CB1R-5-HT2AR heteromer (blue and green protomers) bound to DOI (green surface) and rimonabant (red surface), modeled from the crystal structure of the μ-opioid receptor (PDB id 4DKL) [1]. TMs 5 and 6 of rhodopsin (light brown, 1GZM) [2], the β2-adrenergic receptor (yellow, 2RH1) [3], and the β2-adrenergic receptor in complex with Gs (orange, 3SN6) [4] are superimposed on the 5-HT2AR. This superimposition shows that the conformational equilibrium of GPCRs primarily consists of different conformations of TMs 5 and 6, opening or closing an intracellular cavity for binding of the G-protein with minimal movement of the other TMs. Agonists stabilize conformations of TMs 5 and 6 that facilitate the opening of this intracellular cavity (TMs 5 and 6 in orange), whereas inverse agonists (antagonists) stabilize other conformations of these helices that close this cavity (TMs 5 and 6 in green, light brown, or yellow). TMs 5 and 6 of protomer A, in the closed conformations, can interact with TMs 5 and 6 of protomer B (via a four-helix bundle, green and blue TMs 5 and 6) as observed in the crystal of the μ-opioid receptor. In this assembly, both protomers are locked in the closed conformation since the opening of TMs 5 and 6 for G-protein binding is not feasible. (B) Many GPCRs can bind their G-protein in the absence of an agonist, showing basal activity [5]. This suggests that GPCRs are dynamic proteins that permit rapid small-scale structural fluctuations and pass through an energy landscape to adopt a number of conformations, ranging from inactive to active [6]. The transition probability from one state to another depends on the energy difference between both states and the energy barrier between them. Ligand binding to a monomer (left panel, adapted from [7]) changes the shape of the energy landscape relative to the unliganded landscape (black line), in such a manner that inverse agonists/antagonists (in red) stabilize inactive conformations (red [file pbio.1002194.s008.tif]

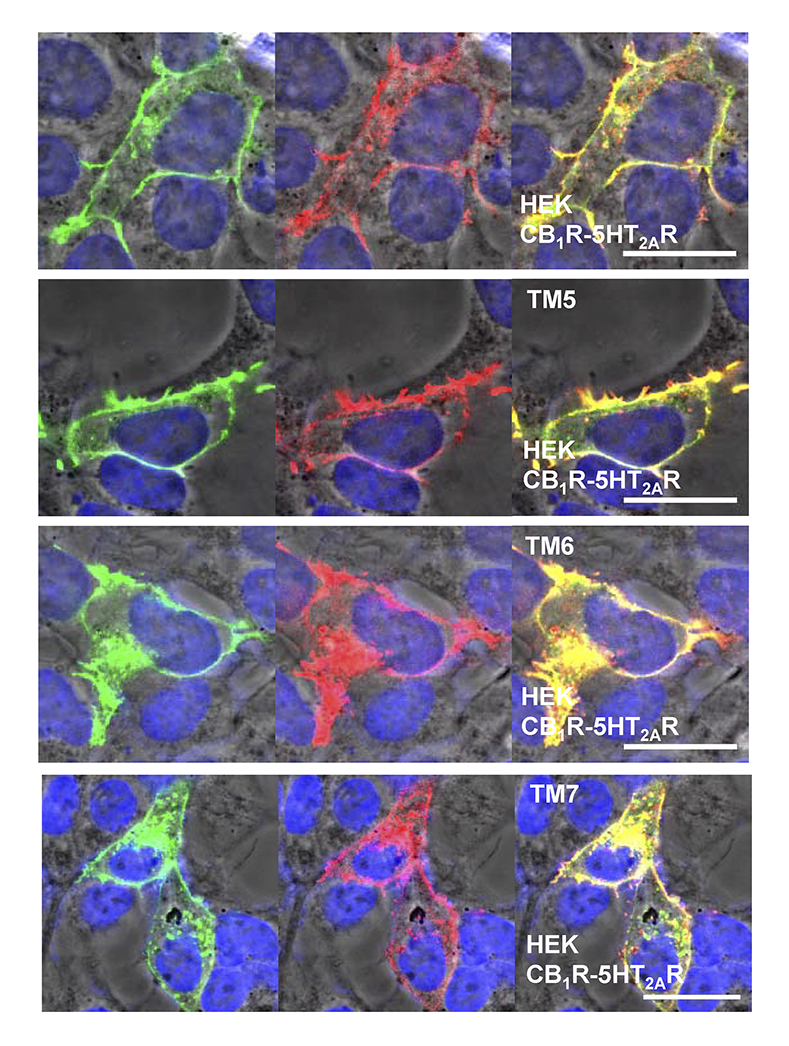

Supplement: S8 Fig — Immunocytochemistry experiments were performed in cells expressing CB1R and 5-HT2AR preincubated for 4 h with vehicle (top panels) or with 4 μM of CB1R TM 5, TM 6, or TM 7 interference peptides using guinea pig anti-CB1R (Frontier Science, Ishikari, Japan) and rabbit anti-5-HT2AR antibody (Neuromics, Edina, Minnesota). Confocal microscopy images showing colocalization (yellow) between CB1R (green) and 5-HT2AR (red) are shown. Scale bars = 20 μm. (TIF) [file pbio.1002194.s009.tif]

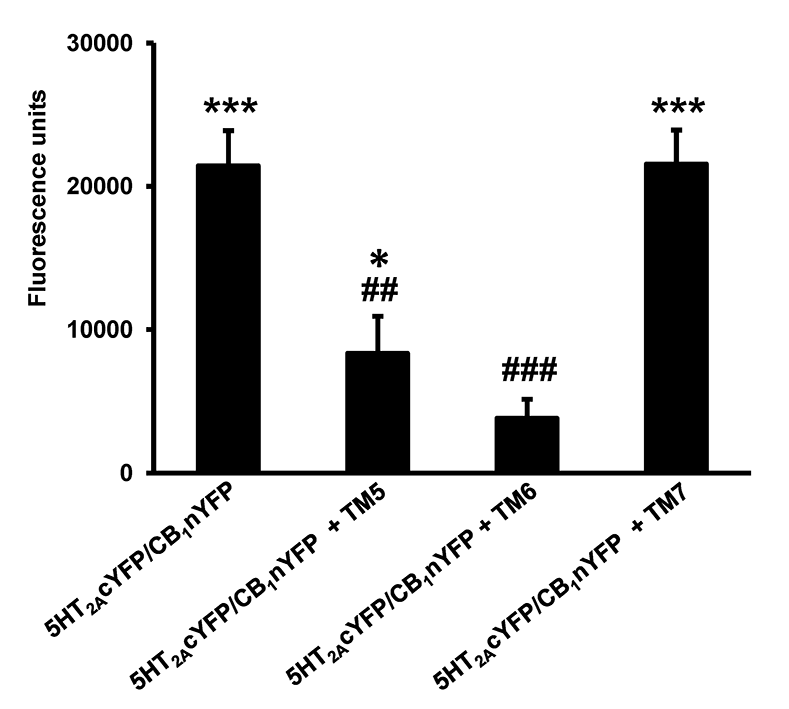

Supplement: S9 Fig — HEK-293T cells transfected with 4 μg of cDNA corresponding to both 5-HT2AR-cYFP and CB1R-nYFP were treated for 4 h with vehicle or 4 μM of CB1R TM 5, TM 6, or TM 7 interference peptides prior to the fluorescence determination at 530 nm. One-way ANOVA followed by Bonferroni post hoc tests showed a significant (* p < 0.05, *** p < 0.001) effect over basal fluorescence (1,500–2,000 fluorescence units in nontransfected cells) or compared to peptide treatment over the vehicle treatment (## p < 0.01, ### p < 0.001). (TIF) [file pbio.1002194.s010.tif]

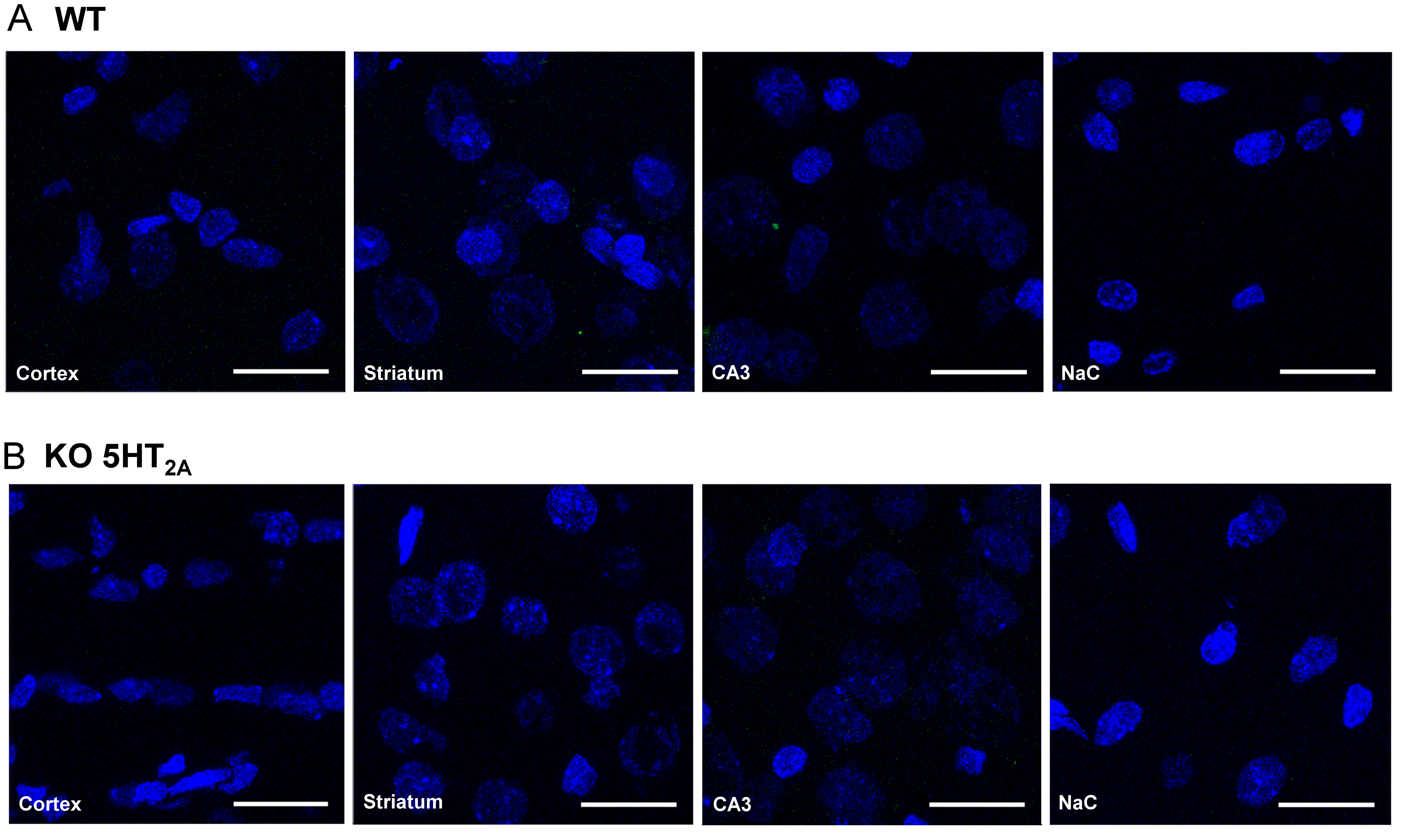

Supplement: S10 Fig — PLAs were performed using slices of mouse cortex (somatomotor layers 1, 2, and 3), caudate-putamen (striatum), hippocampus CA3, or nucleus accumbens (NaC) from WT (A) and 5-HT2AR KO (B) mice, using anti-CB1R and anti-dopamine D1 receptor antibodies as primary antibodies. Confocal microscopy images (superimposed sections) are shown in which green spots corresponding to the heteromers are absent in all panels. In all cases, cell nuclei were stained with DAPI (blue). Scale bars = 20 μm. (TIFF) [file pbio.1002194.s011.tiff]

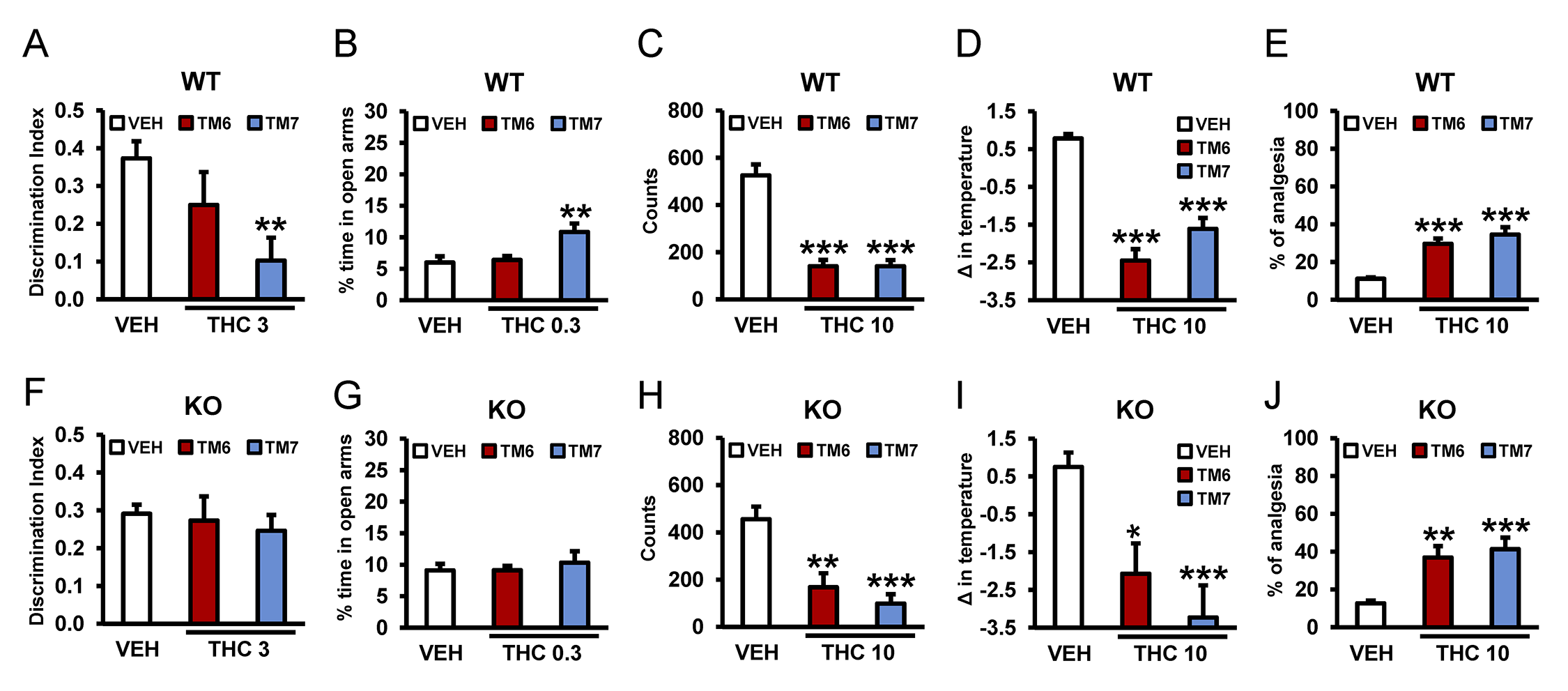

Supplement: S11 Fig — Pretreatment with TM 6, but not with TM 7, peptides (0.2 μg/ 2 μl ICV) blocked the memory deficits and anxiolytic-like behavior induced by THC (3 and 0.3 mg/kg, respectively) in WT mice (A and B), but neither TM 6 nor TM 7 peptides modified these effects in 5-HT2AR KO mice (F and G) (n = 4–6). Hypolocomotion, hypothermia, and analgesia induced by THC (10 mg/kg) were not altered by pretreatment with TM 6 or TM 7 peptides in WT mice (C–E) or in 5-HT2AR KO animals (H–J) (n = 7–11). All data represent mean + SEM. * p < 0.05, ** p < 0.01, *** p < 0.001 versus vehicle. The statistical analyses used and their corresponding F and p values are shown in S2 Table. (TIF) [file pbio.1002194.s012.tif]
