# Supplementary material for: Cognitive Impairment Induced by Delta9-tetrahydrocannabinol Occurs through Heteromers between Cannabinoid CB1 and Serotonin 5-HT2A Receptors
Source: PLoS Biol. 2015 Jul 9;13(7):e1002194. doi: 10.1371/journal.pbio.1002194 (PMC4497644; doi:10.1371/journal.pbio.1002194)
Supplement: S1 Table — Corresponding F and p-values are shown. (DOCX) [file pbio.1002194.s013.docx]

Table S1. Statistical analyses used in animal experiments and their corresponding F and p values.

| **Figure** | **Statistical Test** | **Factor** | **F Value** | | | **p Value** |  | **Figure** | **Statistical Test** | **Factor** | **F Value** | | | **p Value** |
| --- | --- | --- | --- | --- | --- | --- | --- | --- | --- | --- | --- | --- | --- | --- |
| **1A** | Two-way ANOVA | THC | F_(2,44)_ | = | 48.815 | < 0.001 |  | **2E** | Two-way ANOVA | THC | F_(3,99)_ | = | 48.874 | < 0.001 |
|  |  | Genotype | F_(1,44)_ | = | 17.094 | < 0.001 |  |  |  | Genotype | F_(1,99)_ | = | 6.071 | 0.015 |
|  |  | Interaction | F_(2,44)_ | = | 4.348 | 0.019 |  |  |  | Interaction | F_(3,99)_ | = | 2.905 | 0.039 |
| **1B** | Two-way ANOVA | THC | F_(1,38)_ | = | 4.512 | 0.04 |  | **2F** | Two-way ANOVA | THC | F_(1,30)_ | = | 12.773 | 0.001 |
|  |  | Genotype | F_(1,38)_ | = | 0.861 | 0.359 |  |  |  | Genotype | F_(1,30)_ | = | 0.012 | 0.913 |
|  |  | Interaction | F_(1,38)_ | = | 1.532 | 0.223 |  |  |  | Interaction | F_(1,30)_ | = | 0.005 | 0.943 |
| **1C** | Two-way ANOVA | THC | F_(1,21)_ | = | 0.08 | 0.781 |  | **2G** | Three-way ANOVA | Hole | F_(1,25)_ | = | 26.853 | < 0.001 |
|  |  | Genotype | F_(1,21)_ | = | 6.241 | 0.021 |  |  |  | Day | F_(11,275)_ | = | 2.561 | 0.004 |
|  |  | Interaction | F_(1,21)_ | = | 7.619 | 0.012 |  |  |  | Genotype | F_(1,25)_ | = | 0.224 | 0.64 |
| **1E** | One-way ANOVA | THC 1 nM | F_(1,16)_ | = | 5.54 | 0.032 |  |  |  | H x D | F_(11,275)_ | = | 1.739 | 0.065 |
|  |  | THC 10 nM | F_(1,22)_ | = | 0.001 | 0.98 |  |  |  | H x G | F_(1,25)_ | = | 0.166 | 0.687 |
| **1F** | One-way ANOVA | Genotype | F_(1,42)_ | = | 0.654 | 0.423 |  |  |  | D x G | F_(11,275)_ | = | 0.475 | 0.918 |
|  |  |  |  |  |  |  |  |  |  | H x D x G | F_(11,275)_ | = | 1.496 | 0.132 |
| **1G** | Two-way ANOVA | THC | F_(1,28)_ | = | 70.408 | < 0.001 |  | **10A** | Two-way ANOVA | THC | F_(1,29)_ | = | 27.213 | < 0.001 |
|  |  | Genotype | F_(1,28)_ | = | 21.546 | < 0.001 |  |  |  | MDL | F_(1,29)_ | = | 8.216 | 0.008 |
|  |  | Interaction | F_(1,28)_ | = | 13.006 | 0.001 |  |  |  | Interaction | F_(1,29)_ | = | 10.132 | 0.003 |
| **1H** | Two-way ANOVA | THC | F_(1,28)_ | = | 126.38 | < 0.001 |  | **10B** | Two-way ANOVA | THC | F_(1,34)_ | = | 20.792 | < 0.001 |
|  |  | Genotype | F_(1,28)_ | = | 14.112 | 0.001 |  |  |  | Peptides | F_(3,34)_ | = | 2.003 | 0.132 |
|  |  | Interaction | F_(1,28)_ | = | 13.693 | 0.001 |  |  |  | Interaction | F_(3,34)_ | = | 2.878 | 0.05 |
| **1I** | Two-way ANOVA | THC | F_(1,28)_ | = | 125.46 | < 0.001 |  | **10C** | Two-way ANOVA | THC | F_(1,36)_ | = | 4.706 | 0.037 |
|  |  | Genotype | F_(1,28)_ | = | 20.451 | < 0.001 |  |  |  | MDL | F_(1,36)_ | = | 11.251 | 0.002 |
|  |  | Interaction | F_(1,28)_ | = | 12.415 | < 0.001 |  |  |  | Interaction | F_(1,36)_ | = | 7.901 | 0.008 |
| **2A** | Two-way ANOVA | THC | F_(4,90)_ | = | 22.613 | < 0.001 |  | **10D** | Two-way ANOVA | THC | F_(1,29)_ | = | 17.903 | < 0.001 |
|  |  | Genotype | F_(1,90)_ | = | 0.281 | 0.597 |  |  |  | Peptides | F_(3,29)_ | = | 6.614 | 0.002 |
|  |  | Interaction | F_(4,90)_ | = | 0.846 | 0.5 |  |  |  | Interaction | F_(3,29)_ | = | 4.827 | 0.008 |
| **2B** | Two-way ANOVA | THC | F_(3,141)_ | = | 64.702 | < 0.001 |  | **10G** | Two-way ANOVA | THC | F_(1,38)_ | = | 26.588 | < 0.001 |
|  |  | Genotype | F_(1,141)_ | = | 0.001 | 0.977 |  |  |  | Peptides | F_(3,38)_ | = | 2.664 | 0.061 |
|  |  | Interaction | F_(3,141)_ | = | 0.83 | 0.479 |  |  |  | Interaction | F_(3,38)_ | = | 1.878 | 0.15 |
| **2C** | Two-way ANOVA | THC | F_(3,99)_ | = | 43.408 | < 0.001 |  | **10H** | Two-way ANOVA | THC | F_(1,38)_ | = | 32.91 | < 0.001 |
|  |  | Genotype | F_(1,99)_ | = | 0.927 | 0.338 |  |  |  | Peptides | F_(3,38)_ | = | 0.827 | 0.487 |
|  |  | Interaction | F_(3,99)_ | = | 0.614 | 0.607 |  |  |  | Interaction | F_(3,38)_ | = | 0.299 | 0.826 |
| **2D** | Two-way ANOVA | THC | F_(3,95)_ | = | 89.294 | < 0.001 |  | **10I** | Two-way ANOVA | THC | F_(1,38)_ | = | 58.614 | < 0.001 |
|  |  | Genotype | F_(1,95)_ | = | 0.937 | 0.335 |  |  |  | Peptides | F_(3,38)_ | = | 0.448 | 0.72 |
|  |  | Interaction | F_(3,95)_ | = | 1.312 | 0.275 |  |  |  | Interaction | F_(3,38)_ | = | 0.506 | 0.681 |
