# Supplementary material for: Cognitive Impairment Induced by Delta9-tetrahydrocannabinol Occurs through Heteromers between Cannabinoid CB1 and Serotonin 5-HT2A Receptors
Source: PLoS Biol. 2015 Jul 9;13(7):e1002194. doi: 10.1371/journal.pbio.1002194 (PMC4497644; doi:10.1371/journal.pbio.1002194)
Supplement: S2 Table — Corresponding F and p-values are shown. (DOCX) [file pbio.1002194.s014.docx]

Table S2. Statistical analyses used in animal experiments and their corresponding F and p values.

| **Figure** | **Statistical Test** | **Factor** | **F Value** | | | **p Value** |  | **Figure** | **Statistical Test** | **Factor** | **F Value** | | | **p Value** |
| --- | --- | --- | --- | --- | --- | --- | --- | --- | --- | --- | --- | --- | --- | --- |
| **S1B** | Two-way ANOVA | THC | F_(1,19)_ | = | 72.649 | < 0.001 |  | **S11B** | One-way ANOVA | THC | F_(2,24)_ | = | 6.081 | 0.007 |
|  |  | Genotype | F_(1,19)_ | = | 2.741 | 0.114 |  | **S11C** | One-way ANOVA | THC | F_(2,22)_ | = | 37.653 | < 0.001 |
|  |  | Interaction | F_(1,19)_ | = | 0 | 1 |  | **S11D** | One-way ANOVA | THC | F_(2,22)_ | = | 56.188 | < 0.001 |
| **S1C** | Two-way ANOVA | THC | F_(1,19)_ | = | 98.799 | < 0.001 |  | **S11E** | One-way ANOVA | THC | F_(2,22)_ | = | 25.766 | < 0.001 |
|  |  | Genotype | F_(1,19)_ | = | 0.062 | 0.805 |  | **S11F** | One-way ANOVA | THC | F_(2,13)_ | = | 0.268 | 0.769 |
|  |  | Interaction | F_(1,19)_ | = | 2.472 | 0.132 |  | **S11G** | One-way ANOVA | THC | F_(2,13)_ | = | 0.285 | 0.757 |
| **S1D** | One-way ANOVA | Genotype | F_(1,15)_ | = | 6.436 | 0.024 |  | **S11H** | One-way ANOVA | THC | F_(2,13)_ | = | 15.322 | < 0.001 |
| **S1E** | One-way ANOVA | Genotype | F_(1,15)_ | = | 0.007 | 0.935 |  | **S11I** | One-way ANOVA | THC | F_(2,13)_ | = | 9.477 | 0.003 |
| **S11A** | One-way ANOVA | THC | F_(2,23)_ | = | 5.147 | 0.014 |  | **S11J** | One-way ANOVA | THC | F_(2,13)_ | = | 10.866 | 0.002 |
